# Coordination Clusters of 3d-Metals That Behave as Single-Molecule Magnets (SMMs): Synthetic Routes and Strategies

**DOI:** 10.3389/fchem.2018.00461

**Published:** 2018-10-09

**Authors:** Diamantoula Maniaki, Evangelos Pilichos, Spyros P. Perlepes

**Affiliations:** Department of Chemistry, University of Patras, Patras, Greece

**Keywords:** 3d-metal single-molecule magnets, synthetic routes, synthetic strategies, coordination clusters, magnetic properties, reactivity studies, manganese single-molecule magnets, ligand design

## Abstract

The area of 3d-metal coordination clusters that behave as Single-Molecule Magnets (SMMs) is now quite mature within the interdisciplinary field of Molecular Magnetism. This area has created a renaissance in Inorganic Chemistry. From the synthetic Inorganic Chemistry viewpoint, the early years of “try and see” exercises (1993–2000) have been followed by the development of strategies and strict approaches. Our review will first summarize the early synthetic efforts and routes for the preparation of polynuclear 3d-metal SMMs, and it will be then concentrated on the description of the existing strategies. The former involve the combination of appropriate 3d-metal-containing starting materials (simple salts with inorganic anions, metal cardoxylates, and pre-formed carboxylate clusters, metal phosphonates) and one or two primary organic ligands; the importance of the end-on azido group as a ferromagnetic coupler in 3d-metal SMM chemistry will be discussed. The utility of comproportionation reactions and the reductive aggregation route for the construction of manganese SMMs will also be described. Most of the existing strategies for the synthesis of SMMs concern manganese. These involve substitution of carboxylate ligands in pre-formed SMMs by other carboxylate or non-carboxylate groups, reduction procedures for the {${\text{Mn}}_{8}^{\text{III}}$${\text{Mn}}_{4}^{\text{IV}}$} SMMs, spin “tweaking,” “switching on” SMM properties upon conversion of low-spin clusters into high-spin ones, ground-state spin switching and enhancing SMM properties via targeted structural distortions, the use of radical bridging ligands and supramolecular approaches. A very useful strategy is also the “switching on” of SMM behavior through replacement of bridging hydroxide groups by end-on azido or isocyanato ligands in clusters. Selected examples will be mentioned and critically discussed. Particular emphasis will be given on the criteria for the choice of ligands.

## Introduction

Today there is a multi-billion euro industry concerning magnets due to their applications in many useful and modern technologies, such as televisions, credit/debit/ATM cards, motors, computer hard drives, switches and medical equipment, among others. Magnetism has its roots in Ancient Greece; in the province of Magnesia at central Greece, the first discovery of the attraction between lodestone and iron was made. This, however, is a matter of debate. The first observations were made near the city of “Magnesia” (close to the Sipylum mountain) in the old, Asian Minor Greek province of Lydia. This old city was near the modern city Manisa, close to Smirna (Izmir). Sophocles and other Ancient Greek writers reported on the “Lydian stone” in reference to the lodestone (Guimaraes, 2005). There is also a strong piece of evidence that Chinese sailors used the compass. The principles of Magnetism were established through the excellent work of Ampeére, Oersted, Faraday, Maxwell and Pierre Curie, just to name few legendary scientists who contributed in this field of Physics. The discovery of the electron, the seminal ideas of Lewis who wrote simple electronic structures for molecules and ions, and the development of quantum mechanics provided scientists with the appropriate tools for the in-depth understanding of the magnetic properties of the matter. Until ~1960, the popular magnets consisted mainly of metallic alloys and ceramics, e.g., Alnico and ferrites.

Chemists entered the field in the late 50s developing the field of Magnetochemistry. They used experimentally determined magnetic moments and their variation with temperature to draw chemical conclusions and make structural proposals in molecular compounds. Famous examples include: (i) the distinction between the two stereochemistries (tetrahedral, square planar) in 4-coordinate Ni(II) complexes based on the value of the effective magnetic moment, and (ii) the determination by Bleaney and Bowers of the singlet-triplet gap in copper(II) acetate hydrate, before the crystallographic determination of its dinuclear [Cu_2_(O_2_CMe)_4_(H_2_O)_2_] structure.

Molecular Magnetism (Kahn, 1993) came into the scientific scene in the early 80s. It is an interdisciplinary field in which chemists, physicists, material scientists, and chemical engineers, both experimentalists and theoreticians, actively participate. A NATO ASI in Italy in 1983 is widely considered as the beginning of the Molecular Magnetism era. The chemists presented magnetic properties of dinuclear, polynuclear, and polymeric molecular species, whereas the contribution from physicists was based on infinite atom-based arrays with a special focus on 1D systems which were popular at that time (mainly due to the availability of spin Hamiltonians which could be solved). The ASI, which gave the birth of a common scientific language between the two communities, convinced (a) chemists to dare to synthesize complex molecular compounds, and (b) physicists that it was possible to discover new magnetic phenomena working with molecule-based materials (Benelli and Gatteschi, 2015). The development of Molecular Magnetism has been tremendous since then. Milestones include-among others-the synthesis of molecular ferrimagnets, examples of molecular ferromagnets, the discovery that pure organic matter can order ferromagnetically, the attainment of room temperature molecular magnets, the development of spin-crossover systems, the discovery that the magnetic state of several molecular magnets can be photoinduced in a reversible manner, the isolation of systems exhibiting a coexistence of magnetic and other (e.g., conductivity) properties, the development of “magnetic nanowires” (Single-Chain Magnets) and the revolution with Single-Molecule Magnets (Coronado and Gatteschi, 2006; Coronado and Dunbar, 2009; Dunbar, 2012; Palacio, 2014; Benelli and Gatteschi, 2015). Some synthetic aspects of 3d-metal coordination clusters that behave as SMMs are presented in this Review.

An important milestone in the field of Molecular Magnetism was the discovery of the magnetic properties of complex [${\text{Mn}}_{8}^{\text{III}}$${\text{Mn}}_{4}^{\text{IV}}$O_12_(O_2_CMe)_16_(H_2_O)_4_] (**1)** (Caneschi et al., 1991; Sessoli et al., 1993a), which indicated that this molecule can be considered as a small piece of manganese oxide whose further growth is inhibited by the presence of the acetate groups. The molecule possesses 12 spin centers; the 8 Mn^III^ atoms have their spins up and the 4 Mn^IV^ ones down and this coupling scheme result in a *S* = 10 spin ground state (8 × 2 – 4 × 3/2 = 10). The impressive discovery was that, at very low temperatures, the magnetization relaxes slowly and the complex displays hysteresis (of molecular origin) like a tiny magnet. Most remarkably, a negative value for the axial zero-field splitting, *D*, results in the loss of the degeneracy of the corresponding *M*_*S*_ sublevels, and so the *M*_*S*_ = +10 and *M*_*S*_ = −10 levels become lowest in energy. Since the selection rule is Δ*M*_*S*_ = ±1, an energy barrier of *U* = *S*^2^ |*D*| results for “spin up” to “spin down” conversion in the ground state. In the case of **1**, the *S* and *D* values are 10 and −0.5 cm^−1^, respectively; these values give a *U* ≈ 50 cm^−1^ and this results in magnetic blocking below 4 K, as proved by magnetic hysteresis loops. The behavior of **1** gave the idea that information could in the future be stored as the magnetization direction in discrete molecules. Assuming that a diameter for each bit is ~1 nm, a dense array of molecules might achieve a very high surface density (~200,000 gigabits/in^2^), and this is an advantage of several orders of magnitude over the existing magnetic alloy film technologies, a very important consequence for computer hard drives (Beltran and Long, 2005). The ability of a single molecule to behave as a tiny magnet represents another approach, the so-called “bottom-up” approach, to nanoscale magnetic materials, in which the need for smaller and smaller magnetic particles increases continuously (the so-called “top-down” approach). The “bottom-up” route provides this scientific area with many advantages of molecular chemistry, for example good solubility (and not colloid formation observed in magnetic nanoparticles), monodispersity, crystallinity, and avoidance of close contacts between the magnetic cores due to the intervention by an organic shell from the ligands (Bagai and Christou, 2009).

Polynuclear complexes (or coordination clusters) with properties similar to those of **1** are called Single-Molecule Magnets (SMMs); however, some scientists prefer the term Molecular Nanomagnets. In brief, SMMs are individual polynuclear molecules that function as magnets (i.e., they display magnetization hysteresis) below a characteristic blocking temperature, *T*_B_ (which depends on the sweep rate of the magnetic field). *T*_B_ is the highest temperature at which an SMM displays hysteresis in plots of magnetization (*M*) vs. magnetic field (*H*). However, since the *T*_B_ value depends strongly on the sweep rate of *H*, comparisons of blocking temperatures in different SMMs should be done with caution (Woodruff et al., 2013). In the now classical book of Gatteschi, Sessoli, and Villain (Gatteschi et al., 2006), the authors define *T*_B_ as the temperature at which the time for the magnetization relaxation is 100 s. This property arises from a simultaneous presence of a high-spin ground state, *S*, and a large and negative Ising (or easy-axis) type of magnetoanisotropy, as measured by the parameter *D*. This combination gives a significant energy barrier to magnetization reversal (*U)*, the maximum value of which is given by *S*^2^ |*D*| (see above) and (*S*^2^-1/4) |*D*| for integer and non-integer total ground state spins, respectively. However, quantum tunneling of the magnetization vector (QTM) (Friedman et al., 1996; Thomas et al., 1996) through the barrier via *M*_*S*_ levels of higher energy leads to a smaller effective energy barrier (*U*_eff_) than the calculated *U*. In addition to QTM, some SMMs display other interesting quantum properties such as quantum phase interference (Wernsdorfer and Sessoli, 1999; Lecren et al., 2005; Wernsdorfer et al., 2005), exchange-biased QTM (Wernsdorfer et al., 2002; Pinkowicz et al., 2015) and others with potential technological interest (Leuenberger and Loss, 2001; Affronte et al., 2007; Bogani and Wernsdorfer, 2008; Affronte, 2009; Urdampilleta et al., 2011). For all these reasons, SMMs are candidates for a number of specialized applications (Bagai and Christou, 2009). Excellent books (Gatteschi et al., 2006; Benelli and Gatteschi, 2015) and reviews (Gatteschi and Sessoli, 2003; Beltran and Long, 2005; Aromi and Brechin, 2006; Bagai and Christou, 2009; Wang et al., 2011; Milios and Winpenny, 2015) for the properties of transition-metal SMMs are available.

In the last 10 years or so, there has been strong evidences that the most important parameter when designing SMMs is the single-ion anisotropy which should be large. Thus the research focus has been shifted to lanthanides (Ln) and actinides (An) which, in general, have large anisotropies. In Ln(III) SMMs, the required electronic ground-state bistability is due to the [2*J* + 1] *M*_*J*_ microstates within the ground term ^2S+1^L*j* which is spin-orbit-coupled (Woodruff et al., 2013). The advantages of working with An-based SMMs are their large magnetic anisotropy (like the Ln^III^ ions) and the possibility for covalency (like the transition metal ions), advantages which, however, add some complexity (Meinhaus and Long, 2015). Very interesting reviews have been published on the SMM properties of dinuclear and polynuclear Ln(III) (Sessoli and Powell, 2009; Guo et al., 2011; Rinehart and Long, 2011; Habib and Murugesu, 2013; Woodruff et al., 2013; Zhang et al., 2013a,b, 2015; Liddle and van Slageren, 2015; Pedersen et al., 2015; Pointillart et al., 2017; Liu et al., 2018), An (Meinhaus and Long, 2015), 3d/4f (Polyzou et al., 2013; Feltham and Brooker, 2014; Sharples and Collison, 2014; Liu et al., 2015; Rosado Piquer and Sañudo, 2015), and organometallic Ln(III) and An (Layfield, 2014) SMMs.

Before going to the main “menu” of this Review, we would like to say few words about the so-called Single-Ion Magnets (SIMs), which is currently a “hot” research area. These are mainly mononuclear d- or f-metal complexes whose magnetization relaxes slowly not because of cooperative exchange phenomena (as in dinuclear and polynuclear SMMs), but due to the intrinsic properties of the metal ion under an appropriate ligand field. The origin of the barrier here is the magnetic anisotropy. Excellent reviews on transition- (Craig and Murrie, 2015; Bar et al., 2016; Frost et al., 2016), 4f- (Woodruff et al., 2013; Gupta and Murugavel, 2018; Liu et al., 2018) and 5f-metal (Meinhaus and Long, 2015) SIMs are available in the literature. The very recent report of the SIM [(${\text{C}}_{\text{p}}^{\text{ttt}}$)_2_Dy][B(C_6_F_5_)_4_] (**2**) (Goodwin et al., 2017a,b; Guo et al., 2017) with a record *T*_B_ value of ~60 K (*U*_eff_ = 1277 cm^−1^ in zero field) is a revolutionary result, and it predicts a brilliant future in this area; ${\text{C}}_{\text{p}}^{\text{ttt}}$ is the 1,2,4-tri(tert-butyl)cyclopentadienide, i.e., {C_5_H_2_^*t*^Bu_3_-1,2,4} with ^t^Bu = C(Me)_3_. It should be emphasized at this point that the SMM properties of many dinuclear and polynuclear Ln(III) complexes are in fact due to single-ion properties and such molecules can be regarded as a collection of SIMs. This is a consequence of the weak or very weak Ln^III^…Ln^III^ exchange interactions due to the well-known internal character of the 4f orbitals which are severely shielded from the environment; however, most Ln^III^ ions easily produce large anisotropy due to unquenched orbital moment.

The above mentioned developments in the areas of SMMs and SIMs created an explosive growth in synthetic Inorganic (both coordination and organometallic) Chemistry in the first quarter-century of this new “chapter” in the “book” of Molecular Magnetism. New reaction schemes and new types of metal complexes have been discovered. This Review covers the most important (a subjective opinion!) synthetic routes and strategies that have led to SMMs that belong to the family of 3d-metal coordination clusters. This means that synthetic aspects related to SIMs and other types of SMMs (4d-, 5d-, 4f-, 5f-, and d/f-metal-based) are not covered. The content of the Review is purely chemical and it is assumed that the readers have a basic knowledge of structural inorganic chemistry, as well as of the properties of SMMs and the methods of their study. Structural and magnetic descriptions will be confined to the necessary minimum. To avoid long synthetic descriptions, balanced chemical equations (written using molecular-and not ionic-formulae) will be used. In the text, we shall try to explain the synthetic rationale and philosophy behind the reactions with particular emphasis on the choice of reactants (ligands and metal-containing starting materials). Synthetic schemes, routes and strategies for the preparation of 3d-metal SMMs have appeared in several books, reviews, and papers along with detailed structural and magnetic descriptions; this is, however, the first review which is concentrated exclusively on the synthetic and reactivity chemistry of 3d-metal SMMs. Since there are thousands of papers describing the syntheses and properties of polynuclear 3d-metals SMMs, it is inevitable that we cannot cover all the existing literature. Thus, we express our apologies to the scientists whose excellent research will not be cited here.

The popular Harris notation (Coxall et al., 2000) will be used to describe the ligands' coordination modes. The Harris notation describes the ligation mode as X.Y_1_Y_2_Y_3_…Y_n_, where X is the total number of metal centers bound by the ligand, and each of Y refers to the number of metal ions attached to the different donor atoms. The order of the Y groups follows the well-known Cahn-Ingold-Prelog priority rules; therefore, for the most ligands reported in this work O comes before N. From time to time the traditional η/μ notation will be also used.

## Synthesis of 3d-metal SMMs: general considerations

The 3d-metal systems that have been used in the preparation of SMMs are V(III), V(IV), Mn(II/III/IV), Mn(III), Mn(II/III), Mn(III/IV), Fe(II), Fe(III), Co(II), Co(II/III), Ni(II), and Cu(II). The most effective and well-studied SMMs are those containing high-spin Mn(III). The presence of high-spin octahedral Mn^III^ atoms in molecules is valuable for making SMMs; the reason is that this 3d^4^, Jahn-Teller elongated ion has a large easy-axis anisotropy (negative *D*) which is desirable. Thus, many of the routes and strategies below involve examples from Mn chemistry.

**Figure 1:**
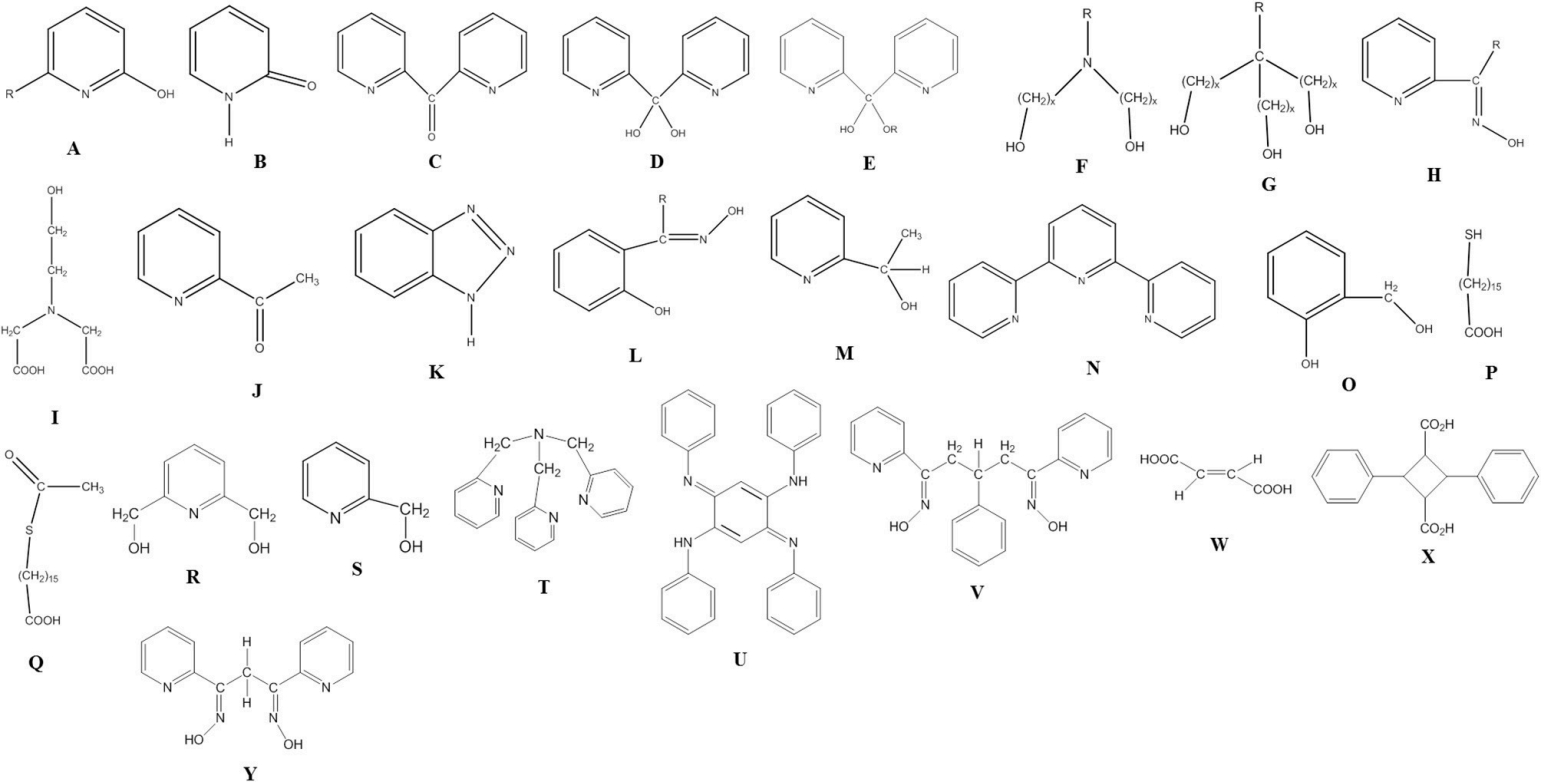
The structural formulae of some of the organic ligands discussed in this review (R = various).

All 3d-metal SMMs are dinuclear or polynuclear complexes. We are doing a parenthesis here to mention that the exchange coupling, *J*, is an important parameter to consider in the preparation of SMMs. This should be strong, because otherwise the spin ground state is not energetically isolated. Single-molecule magnetism is a ground-state phenomenon, and thus in order SMM properties to be observed at a certain temperature, the ground state must be ideally occupied at this temperature. The “translation” of this is that the bridging ligand or ligands should propagate strong ferromagnetic exchange interactions between the metal centers resulting in high-spin ground states (large *S* values). However, large *S* values can also arise from competing antiferromagnetic interactions in some topologies that prevent (“frustrate”) exactly antiparallel spin alignments. Organic ligands discussed in this Review are shown in Figure 1. Many of these ligands can provide monoatomic bridges which often promote ferromagnetic exchange interactions.

A great development in synthetic chemistry during the twentieth century has been the ability of organic chemists to make large, complicated molecules in a systematic, step by step controlled manner, based on the known behavior of carbon and well-established reaction patterns. Sometimes, three or more different parts of a molecule are synthesized separately before the whole species is assembled in two or three final steps. Unfortunately, this is not possible for the transition-metal synthetic chemists, whose efforts are complicated by the non-predictable nature of the metal (e.g., variable oxidation state and coordination number), and the influence of reaction stoichiometry and solvent on product identity. For this reason, many synthetic schemes for SMMs are based on one- or two-step reactions. The lack of control in transition-metal chemistry has led to the adoption of the neologism “self-assembly” (Winpenny, 1999). The situation is becoming worse for the inorganic chemist who must crystallize her/his products (and SMM candidates) and determine their structures by single-crystal X-ray crystallography; this is often a difficult task. On the contrary, organic chemists can use all the “arsenal” of NMR and mass spectroscopic methods (which have undergone an explosive growth in lase decades) to firmly establish the structures, with no need to crystallize the compounds.

## The years of innocence: “try and see” exercises

The discovery of the exciting magnetic properties of **1** ignited many efforts to synthesize dinuclear and polynuclear (coordination clusters) 3d-metal complexes with SMM properties. Most research groups, including our group, used simple 3d-metal “salts” with coordinating (e.g., Cl^−^, Br^−^, ${\text{NO}}_{3}^{-}$, SCN^−^, ${\text{SO}}_{4}^{2-}$,…) or non-coordinating (e.g., ${\text{ClO}}_{4}^{-}$, ${\text{PF}}_{6}^{-}$, ${\text{BF}}_{4}^{-}$,…) inorganic anions and flexible polydentate organic ligands. Such reaction schemes permit a “conversation” between the preferred coordination geometry of the metal ion and the coordination mode of the ligand. If exclusive formation of 5- or 6-membered chelating rings is avoided, the coordinative flexibility of the polydentate ligand is large. This allows the isolation of coordination clusters with a variety of unpredictable structures (and hopefully SMM properties), almost always incorporating further auxiliary bridging groups formed *in situ*, e.g., OH^−^, RO^−^, O^2−^, depending on the solvent and the “pH” of the reaction medium. Organic ligands possessing one, two, three or more ionizable hydrogens exhibit better bridging capabilities, if fully or in part deprotonated (with the help of an external base). The fate of the inorganic anion, present in the 3d-metal starting material, depends on the reaction conditions. It can participate in the cluster (either as a ligand or a counterion) or it can be absent. If there is evidence (or suspicion) for the presence of anionic cluster species in solution, bulky cations, e.g., Bu^*n*^_4_N^+^, should be used to precipitate the product.

In a number of reaction schemes, the 3d-metal starting material contains a strongly basic anionic group, e.g., a carboxylate group (${\text{MeCO}}_{2}^{-}$, ${\text{PhCO}}_{2}^{-}$,…) or a β-diketonate ion (acac^−^, bzac^−^, dbm^−^,…), where Me = methyl, Ph = phenyl, acacH = acetylacetone, bzacH = benzoylacetone, and dbmH = dibenzoylmethane. If the primary organic ligand LH_x_ (x = 1,2,3,…) is not in excess, then it can be deprotonated by the auxiliary organic anion while an amount of the latter remains in solution. This organic “blend,” which contains the deprotonated primary organic ligand and the anionic auxiliary organic group, leads often to dinuclear or polynuclear 3d-metal complexes which often exhibit interesting magnetic properties. This route is particularly useful when external bases for ligand deprotonation perplex the reaction, e.g., by precipitating amorphous hydroxido- or/and oxido-containing materials.

It would be naïve to believe that the above described “try and see” approach leads directly to SMMs. Considerable forethought and hindsight in the metal ions, their inorganic or auxiliary organic anions, primary organic ligands and reaction conditions (reaction ratio, solvent, “pH,” nature of external base, temperature, pressure, crystallization technique, etc.) are necessary to “switch on” or improve the SMM properties in the products.

The idea of preparing coordination clusters without strictly designing the product has been termed “serendipitous assembly” (Winpenny, 2002) and it is a productive means of preparing SMMs. The seminal review-type article by Winpenny has made inorganic chemists guiltless of the “crime,” the crime being their ability to isolate complexes with exciting molecular structures and impressive magnetic properties within few days!

Before proceeding to representative examples of the “try and see” route, we would like to mention that among the large number of primary organic ligands used in this chemistry are 2-hydroxypyridines (**A**)/2-pyridones (**B**) (Brechin et al., 1999; Winpenny, 2002, 2004), di-2-pyridyl ketone (**C**) and its related *gem*-diol (**D)** and hemiketal (**E**) ligands (Papaefstathiou and Perlepes, 2002; Stamatatos et al., 2009), amino diol-type ligands (**F**) (Tasiopoulos and Perlepes, 2008), tripodal alcohols (**G**) (Brechin, 2005), 2-pyridyl oximes (**H**) and related ligands (Milios et al., 2006), mixed hydroxyl-carboxylic acid ligands (e.g., **I**) (Powell et al., 1995), shown in Figure 1, and myriads of polydentate Schiff-base ligands (Hernández-Molina and Mederos, 2004).

A final point of general discussion is the importance of the presence of terminal ligands in the reaction media; these are necessary for the termination of a possible polymerization process which would lead to 1D, 2D, or 3D coordination polymers. In the chemistry of 3d-metal coordination clusters, this role is often fulfilled by the solvent molecules, the inorganic anions of the 3d-metal starting materials, the auxiliary organic ligands and the “chelating” part of the bridging organic ligands, or by the purposefully addition of a chelating ligand in the reaction systems.

### Simple 3d-metal starting materials with inorganic anions

**Figure 2:**
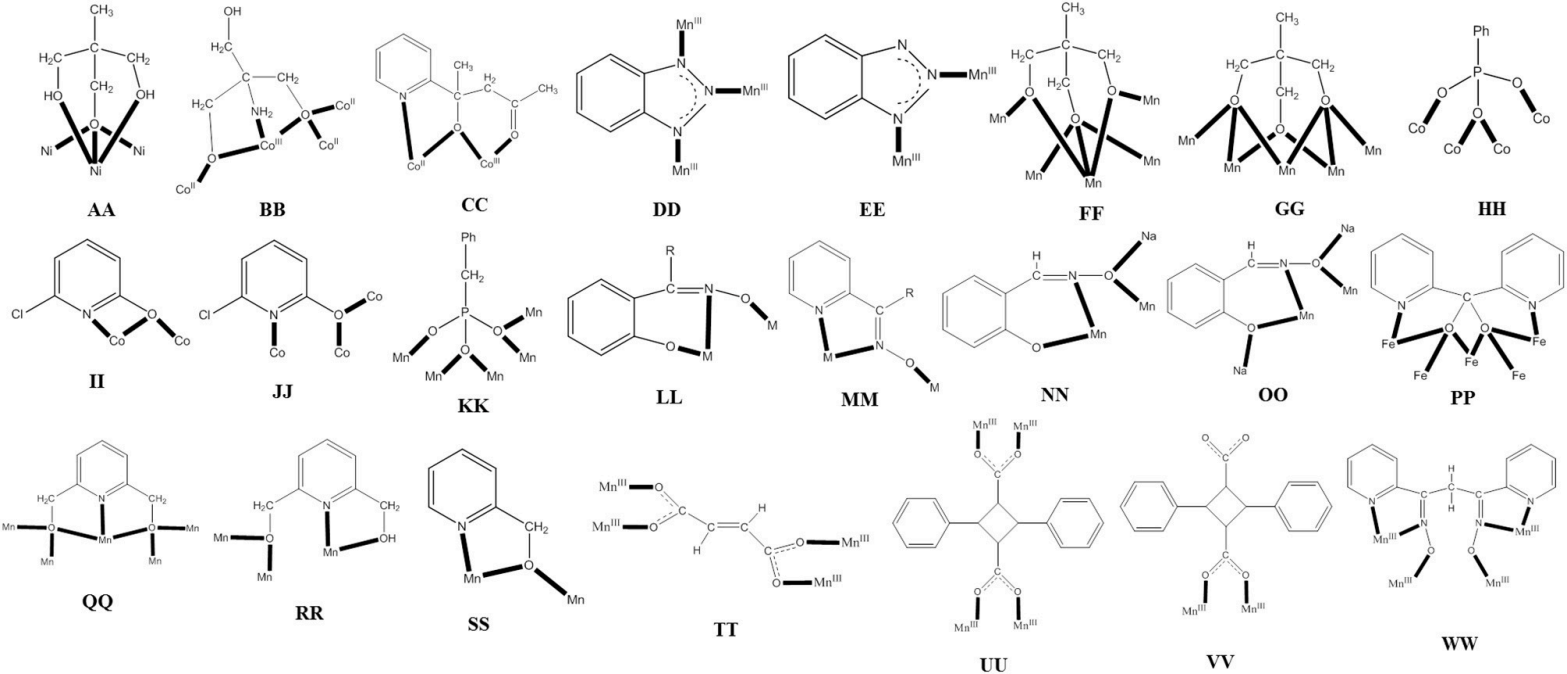
The coordination modes of some ligands discussed in this review; see the text for further details.

Commonly used 3d-metal “salts” with inorganic anions include halides, nitrates, sulfates, perchlorates, etc. Ni(II) is promizing in the synthesis of SMMs due to its significant single-ion anisotropy. For example, the reaction between Ni(NO_3_)_2_ ·6H_2_O, 1,1,1-tris(hydroxymethyl)ethane (H_3_thme; **G** with × = 1 and R = Me in Figure 1) and NaOMe in MeOH gives complex [Ni_4_(H_2_thme)_4_(MeCN)_4_](NO_3_)_4_ (**3**), Equation (1) (Moragues-Cánovas et al., 2004). The initial reaction solution was evaporated to give a solid residue that was extracted with MeCN to give blue crystals of the product. The cation has a cubane structure. Each Ni^II^ atom is surrounded by two neutral oxygen atoms of H_2_thme^−^ that act as terminal donors and three alkoxido-like deprotonated oxygen atoms from three ligands; thus, each monoanion behaves as a 3.311 ligand (**AA** in Figure 2); one MeCN molecule completes six coordination at each metal center. Intramolecular ferromagnetic Ni^II^…Ni^II^ exchange interactions give an *S* = 4 ground state which is 40 cm^−1^ lower in energy from the first *S* = 3 excited state. Single-crystal magnetization vs. field (*H*) measurements reveal an SMM behavior below 0.5 K with fast magnetization relaxation due to QTM. The width of the hysteresis loop depends on the “history” of the sample, i.e., the starting magnetic state before varying the magnetic field.

(1) $$\begin{array}{l} 4\text{ Ni}{\left({\text{NO}}_{3}\right)}_{2}\;\cdot 6{\text{H}}_{2}\text{O}+4\;{\text{H}}_{3}\text{thme}+4\text{ NaOMe}\\ \;+4\text{ MeCN}\overset{\text{MeOH}/\text{MeCN}}{\to }\left[{\text{Ni}}_{4}{\left({\text{H}}_{2}\text{thme}\right)}_{4}{\left(\text{MeCN}\right)}_{4}\right]{\left({\text{NO}}_{3}\right)}_{4}\\ \;+4\;{\text{NaNO}}_{3}+4\text{ MeOH}+24\;{\text{H}}_{2}\text{O} \end{array}$$

Another member of the ligand family **G** (see Figure 1), but in Co chemistry this time, gives a structurally and magnetically interesting mixed-valence coordination cluster. The *D* parameter for the Co^II^ single ion in a tetragonally distorted octahedral environment is positive, because the *M* = ±1/2 ground state is lower in energy than the *M* = ±3/2 state, these two states being the Kramers' doublets that derive from the ^4^A_2g_ ground state in ideal D_4h_ symmetry due to spin-orbit coupling However, the orientation of the axial zero-field tensors on the Co^II^ atoms, that are part of a cluster, may be favorable with respect to the molecular easy axis giving an overall negative *D*-value (Ising type anisotropy) and leading to SMM behaviors (Kostakis et al., 2012). The 1:1 reaction between Co(NO_3_)_2_ · 6H_2_O and 2-amino-2(hydroxymethyl)propane-1,3-diol (H_3_ahmpd; **G** with R = NH_2_ in Figure 1) in H_2_O, followed by increase of the pH to 7.5 by addition of (Bu^*n*^_4_N)OH· 5H_2_O gives a solution; removal of water under vacuum and dissolution of the residue in MeOH leads to cluster [${\text{Co}}_{4}^{\text{II}}$${\text{Co}}_{3}^{\text{III}}$(Hahmpd)_6_(NO_3_)_3_(H_2_O)_3_](NO_3_)_2_ (**4**), Equation (2) (Ferguson et al., 2007). The yield is very low (~5%) but the reaction is reproducible. The cation is disk-like with the octahedral metal ions approximately coplanar and bridged exclusively by the alkoxido groups of the doubly deprotonated ligands, which adopt the 4.3201 coordination mode (**BB** in Figure 2). One alkoxido oxygen atom of each ligand is μ_3_, bridging the central Co^II^ to one outer Co^II^ and one Co^III^, with the second alkoxido oxygen bridging one outer Co^II^ to a Co^III^ center; the amine group is bound to Co^III^. The coordination at each outer Co^II^ is completed by a H_2_O molecule and a monodentate nitrato group. The complex is an exchange-biased SMM. For a well-isolated SMM, the first step in the hysteresis loop due to QTM should occur at *H* = 0 T. For **4**, due to weak intermolecular interactions, this step is shifted to ±30 mT, suggesting a small antiferromagnetic exchange bias between cations of about 30 mT.

(2) $$\begin{array}{l} 7\;{\text{Co}}^{\text{II}}{\left({\text{NO}}_{3}\right)}_{2}\cdot 6{\text{H}}_{2}\text{O}+6\;{\text{H}}_{3}\text{ahmpd}+9\;\left({\text{Bu}}_{\;4}^{\text{n}}\text{N}\right)\text{OH}\cdot 5{\text{H}}_{2}\text{O}\\ \;+3/4{\text{O}}_{2}\overset{{\text{H}}_{2}\text{O}}{\to }\left[{\text{Co}}_{4}^{\text{II}}{\text{Co}}_{3}^{\text{III}}{\left(\text{Hahmpd}\right)}_{6}{\left({\text{NO}}_{3}\right)}_{3}{\left({\text{H}}_{2}\text{O}\right)}_{3}\right]{\left({\text{NO}}_{3}\right)}_{2}\\ \;+9\;\left({\text{Bu}}_{\;4}^{\text{n}}\text{N}\right)\left({\text{NO}}_{3}\right)+94.5\;{\text{H}}_{2}\text{O} \end{array}$$

**Figure 3:**
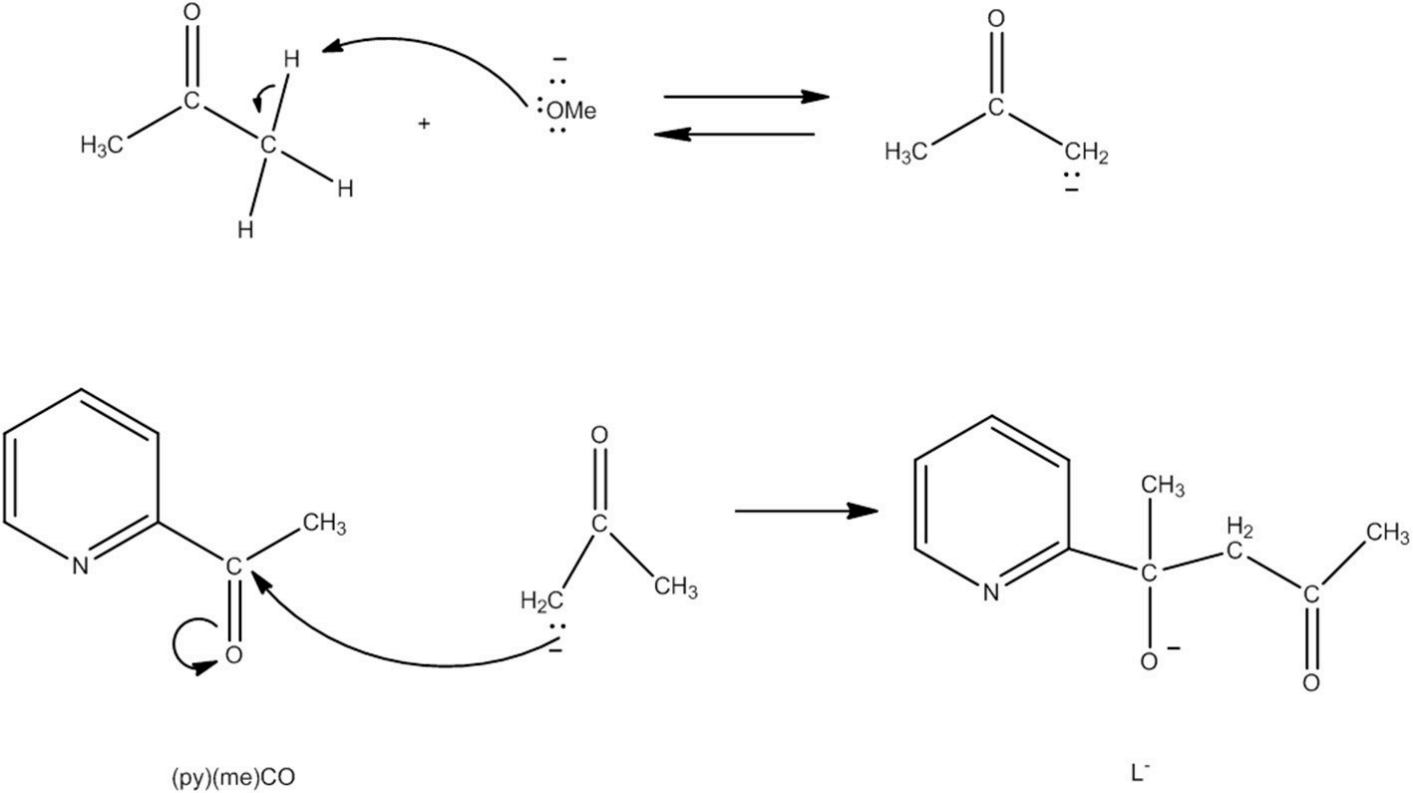
Simplified view of the mechanism that gives the ligand L^−^ which participates in complex **5** (Schematic drawing inspired by Kitos et al., 2011).

Several Co_7_ coordination clusters have a wheel-shaped (or disk-like) metal topology similar to that found in **4** (Kitos et al., 2011). The metal oxidation levels in these compounds are ${\text{Co}}_{7}^{\text{II}}$, ${\text{Co}}_{6}^{\text{II}}$Co^III^, ${\text{Co}}_{3}^{\text{II}}$${\text{Co}}_{4}^{\text{III}}$, and ${\text{Co}}_{4}^{\text{II}}$${\text{Co}}_{3}^{\text{III}}$; few of them are SMMs. In another example (Kitos et al., 2011), the reaction of Co(ClO_4_)_2_ · 6H_2_O and 2-acetylpyridine [(py)(me)CO; **J** in Figure 1] in the presence of (Bu^*n*^_4_N)OMe (~1:1:1) in Me_2_CO at room temperature under aerobic conditions gave a brown solution, from which the green compound [${\text{Co}}_{6}^{\text{II}}$Co^III^(OH)_6_(L)_6_](ClO_4_)_3_ (**5**) was isolated in a moderate yield, Equation (3). The ligand L^−^ which participates in the complex is the anion of 2-(pyridine-2-yl)pentane-2-ol-4-one, (py)(me)C(CH_2_COCH_3_)(O)^−^, formed in solution (*in situ*) through a metal ion-assisted, crossed-aldol reaction in Me_2_CO under strongly basic conditions (see Figure 3). The disk-like cation of **5** constists of a central octahedral Co^III^ atom linked to six peripheral Co^II^ atoms (also octahedral) by six 3.3 hydroxido groups; the six Co^II^ centers on the rim are held together by six 2.211 L^−^ ligands (**CC** in Figure 2) and the oxygen atoms of the hydroxido groups. Each heptanuclear cation is a weak SMM but there are also intermolecular interactions (proven by crystallography) leading to a 3D architecture; this gives magnetization hysteresis at 5 K.

(3) $$\begin{array}{l} 7\;{\text{Co}}^{\text{II}}{\left({\text{ClO}}_{4}\right)}_{2}\cdot 6{\text{H}}_{2}\text{O}+6\left(\text{py}\right)\left(\text{me}\right)\text{CO}+11\;\left({\text{Bu}}_{\;4}^{\text{n}}\text{N}\right)\text{OMe}\\ \;+6\;{\text{CH}}_{3}{\text{COCH}}_{3}+¼{\text{O}}_{2}\overset{{\text{Me}}_{2}\text{CO}}{\to }[{\text{Co}}_{6}^{\text{II}}{\text{Co}}^{\text{III}}{\left(\text{OH}\right)}_{6}\{\left(\text{py}\right)\left(\text{me}\right)\;\\ \;\text{C}\left({\text{CH}}_{2}{\text{COCH}}_{3}\right)\left(\text{O}\right){\}}_{6}]{\left({\text{ClO}}_{4}\right)}_{3}+11\;\left({\text{Bu}}_{\;4}^{\text{n}}\text{N}\right)({\text{ClO}}_{4})\\ \;+11\text{ MeOH}+36.5\;{\text{H}}_{2}\text{O} \end{array}$$

When 3d-metal fluorides are used as starting materials in SMM chemistry, their insolubility in common organic solvents is a problem. In order to overcome this disadvantage, “melt” reactions are often used. For example, Collison, Brechin, and co-workers (Jones et al., 2004) mixed together and heated MnF_3_ and benzotriazole (Hbta; **K** in Figure 1), under nitrogen, to the melting point of the organic ligand (100°C). As the ligand melts, the MnF_3_ dissolved and the reaction takes place. After drying of the resulting black “melt” under vacuum and extraction of the solid mixture into MeOH at 50°C, black crystals of [${\text{Mn}}_{26}^{\text{III}}$O_17_(OH)_8_(OMe)_4_(bta)_22_(MeOH)_14_(H_2_O)_2_] (**6**) are obtained in a low yield (~10%), Equation (4). The molecule of **6** consists of an array of Mn^III^ tetrahedra linked by 3.3-O^2−^ ions, 3.3- and 3.2-OH^−^ ions, 2.2-MeO^−^ ions, and 3.111 and 2.110 bta^−^ ligands (**DD** and **EE** in Figure 2). Single-crystal magnetic studies at low temperatures performed on **6** (*S* = 4, *D* = −0.90 cm^−1^) revealed the time- and temperature-dependent magnetization hysteresis loops indicative of SMM behavior. The SMM parameters are *U*_eff_ = 10.4 cm^−1^ and τ_0_ = 3 × 10^−9^ s. Below ~0.2 K, there is strong evidence for the presence of QTM in the ground state because the relaxation rates are independent of the temperature.

(4) $$\begin{array}{l} 26\;{\text{Mn}}^{\text{III}}{\text{F}}_{3}+22\text{ Hbta}+18\text{ MeOH}\\ \;+27\;{\text{H}}_{2}\text{O}\overset{\text{MeOH}}{\underset{\text{T}}{\to }}[{\text{Mn}}_{26}^{\text{III}}{\text{O}}_{17}{\left(\text{OH}\right)}_{8}{\left(\text{OMe}\right)}_{4}{\text{F}}_{10}{\left(\text{bta}\right)}_{22}\\ \;{\left(\text{MeOH}\right)}_{14}{\left({\text{H}}_{2}\text{O}\right)}_{2}]+68\text{ HF} \end{array}$$

### Simple 3d-metal carboxylates and carboxylate triangles and butterflies as starting materials

Simple 3d-metal carboxylates are very popular in the synthesis of coordination clusters and SMMs, especially when the primary organic ligand possesses ionizable hydrogens. If the carboxylate to primary organic ligand reaction ratio is large enough, carboxylate groups are involved in the products either as terminal or (more often) as bridging ligands. We give two examples of Mn SMMs prepared using this route.

Complex [${\text{Mn}}_{9}^{\text{III}}$O_4_(OMe)_4_(O_2_CMe)_3_(mesao)_6_(H_2_O)_2_] (**7**) can be prepared successfully (and in a serendipitous way) by adding a variety of highly charged anions or cations to a reaction solution comprising Mn(O_2_CMe)_2_·4H_2_O, mesaoH_2_ and Et_3_N in MeOH, Equation (5); mesao^2−^ is the dianion of the R = Me derivative of salicylaldoxime (**L** in Figure 1). Equivalent reactions without the presence of highly charged anions or cations affords hexanuclear Mn(III) complexes. It has been suggested (Inglis et al., 2011) that these ions simply enforce changes in the self-assembly process (thus avoiding the ${\text{Mn}}_{6}^{\text{III}}$ clusters) by their mere presence in the reaction mixtures. The metal topology can be described as a partial supertetrahedron in which the upper vertex is absent. The complex is an SMM with a *U*_eff_ value of ~21 cm^−1^ and-at the time of publication- represented the first chiral SMM obtained from achiral starting materials. Fitting of the dc magnetization data give the parameters *S* = 6, *g* = 1.98, and *D* = −0.60 cm^−1^.

(5) $$\begin{array}{l} 9\;{\text{Mn}}^{\text{II}}{\left({\text{O}}_{2}\text{CMe}\right)}_{2}\cdot 4{\text{H}}_{2}\text{O}+6\;{\text{mesaoH}}_{2}+4\text{ MeOH}+15\;{\text{Et}}_{3}\text{N}\\ \;+9/4{\text{O}}_{2}\overset{\text{MeOH}/\text{MeCN}}{\underset{\text{Ln}{\left({\text{NO}}_{3}\right)}_{3}\cdot 6{\text{H}}_{2}\text{O}}{\to }}[{\text{Mn}}_{9}^{\text{III}}{\text{O}}_{4}{\left(\text{OMe}\right)}_{4}{\left({\text{O}}_{2}\text{CMe}\right)}_{3}{\left(\text{mesao}\right)}_{6}\\ \;{\left({\text{H}}_{2}\text{O}\right)}_{2}]+15\;({\text{Et}}_{3}\text{NH})\left({\text{O}}_{2}\text{CMe}\right)+34.5\;{\text{H}}_{2}\text{O} \end{array}$$

The use of α-methyl-2-pyridine-methanol (mpmH; **M** in Figure 1) in Mn carboxylate chemistry has provided access to the first {Mn_31_} coordination cluster (Abasi et al., 2017). The 2:1:2 Mn(O_2_CPh)_2_·2H_2_O/rac-mpmH/Et_3_N reaction mixture in MeOH gives a dark red solution, from which complex [${\text{Mn}}_{2}^{\text{II}}$${\text{Mn}}_{28}^{\text{III}}$Mn^IV^O_24_(OH)_2_(OMe)_24_(O_2_CPh)_16_(rac-mpm)_2_] (**8**) can be crystallized in 30% yield, Equation (6). The core can be described as a consecutive array of edge-sharing {Mn_4_(μ_4_-O)} tetrahedra and {Mn_3_(μ_3_-O)} triangles that are connected to each other via bridging oxido and methoxido groups. Complex **8** is not only structurally novel due to its nano-size dimensions, but also behaves as SMM with one of the largest *U*_eff_ values (~40 cm^−1^) yet reported for a 3d-metal SMM; moreover, it presents magnetization loops below 5 K, one of the highest *T*_B_ temperatures at which molecular hysteresis has been observed for 3d-metal-based SMMs. Ac magnetic susceptibility measurements indicate an *S* = 23/2 ground state for this giant cluster.

(6) $$\begin{array}{l} 31\text{ Mn}{\left({\text{O}}_{2}\text{CPh}\right)}_{2}\cdot 2{\text{H}}_{2}\text{O}+2\text{ rac​​-​​mpmH}+46\;{\text{Et}}_{3}\text{N}+24\text{ MeOH}\\ \;+15/2\;{\text{O}}_{2}\overset{\text{MeOH}}{\to }[{\text{Mn}}_{2}^{\text{II}}{\text{Mn}}_{28}^{\text{III}}{\text{Mn}}^{\text{IV}}{\text{O}}_{24}{\left(\text{OH}\right)}_{2}{\left(\text{OMe}\right)}_{24}\\ \;{\left({\text{O}}_{2}\text{CPh}\right)}_{16}{\left(\text{rac​​}-\text{​​mpm}\right)}_{2}]+46\;\left({\text{Et}}_{3}\text{NH}\right)\;({\text{O}}_{2}\text{CPh})+51\;{\text{H}}_{2}\text{O} \end{array}$$

A common synthetic route for preparing Mn coordination clusters and SMMs is the reaction of a pre-formed carboxylate cluster with a polydentate organic ligands (either bridging or chelating). Two frequently used Mn(II) carboxylate sources are the family of triangular oxido-centered complexes of the general formulation [Mn_3_O(O_2_CR)_6_L_3_]^0,+^ (Vincent et al., 1987), where L is a neutral monodentate ligand or solvent and R = Me, Ph,…, and the butterfly anionic complex (Bu^*n*^_4_N)[${\text{Mn}}_{4}^{\text{III}}$O_2_(O_2_CPh)_9_(H_2_O)] (Wang et al., 1991). Two examples (from hundreds in the literature) are illustrated below.

The reaction of [${\text{Mn}}_{3}^{\text{II},\text{III},\text{III}}$O(O_2_CPh)_6_(py)_3_] (py = pyridine) and 1,1,1-tris(hydroxymethyl)ethane (H_3_thme; **G** with x = 1 and R = Me in Figure 1) in MeCN gives complex [${\text{Mn}}_{2}^{\text{II}}$${\text{Mn}}_{10}^{\text{III}}$O_4_(OH)_2_(O_2_CPh)_12_(thme)_4_(py)_2_] (**9**) in 20% yield, Equation (7). The molecule has a rod- or ladder-like structure and its core can be described as consisting of 10 edge-sharing {Mn_3_(μ_3_-O)} triangles or five edge-sharing {Mn_4_(μ_3_-O)_2_} units. The four triply deprotonated ligands sit above and below the {${\text{Mn}}_{2}^{\text{II}}$${\text{Mn}}_{10}^{\text{III}}$O_4_(OH)_2_} plane and are two types: two use two of their arms in a μ_2_-fashion with the third arm acting as a μ_3_-bridge adopting the 5.322 coordination mode (**FF** in Figure 2); the other two thme^3−^ ligands have two μ_3_-arms and one μ_2_-arm adopting the 5.332 coordination mode (**GG** in Figure 2). The complex has an *S* = 7 ground state with *g* = 1.98 and *D* = −0.09 cm^−1^. There are low-lying excited states with *S* values greater than the *S* = 7 ground state. The appearance of single-crystal magnetization hysteresis loops and relaxation measurements shows this complex to be an SMM (Rajaraman et al., 2004).

(7) $$\begin{array}{l} 4\;\left[{\text{Mn}}_{3}^{\text{II,III,III}}\text{O}{\left({\text{O}}_{2}\text{CPh}\right)}_{6}{\left(\text{py}\right)}_{3}\right]+4\;{\text{H}}_{3}\text{thme}+{\text{H}}_{2}\text{O}\\ \;+1/2\;{\text{O}}_{2}\;\overset{\text{MeCN}}{\to }[{\text{Mn}}_{2}^{\text{II}}{\text{Mn}}_{10}^{\text{III}}{\text{O}}_{4}{\left(\text{OH}\right)}_{2}{\left({\text{O}}_{2}\text{CPh}\right)}_{12}{\left(\text{thme}\right)}_{4}\\ \;{\left(\text{py}\right)}_{2}]+10\;\left(\text{pyH}\right)\left({\text{O}}_{2}\text{CPh}\right)+2\;{\text{PhCO}}_{2}\text{H} \end{array}$$

**Figure 4:**
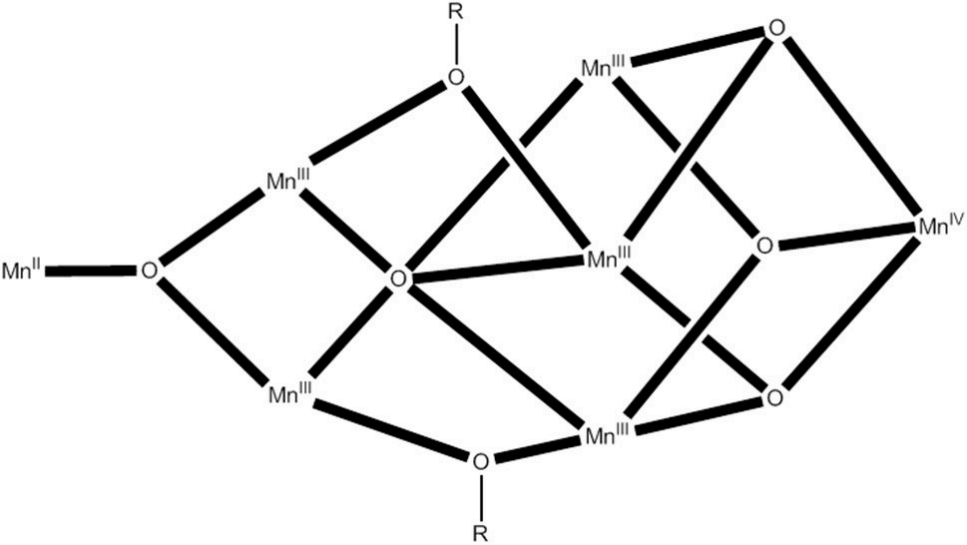
The novel manganese-oxygen core of complex **10**; coordination bonds are drawn in bold. R– = PhCH_2_- (Schematic drawing inspired by Mishra et al., 2006).

The (Bu^*n*^_4_N)[${\text{Mn}}_{4}^{\text{III}}$O_2_(O_2_CPh)_9_(H_2_O)]/terpy (2.5:1) reaction mixture in MeCN/PhCH_2_OH gives a brown solution, from which complex [Mn^II^${\text{Mn}}_{5}^{\text{III}}$Mn^IV^O_5_(OCH_2_Ph)_2_(O_2_CPh)_9_(terpy)] (**10**) is subsequently isolated in ~50% yield, Equation (8); terpy is the rigid tridentate chelating ligand 2,2′:6′, 2″-terpypyridine (**N** in Figure 1) (Mishra et al., 2006). A Mn: terpy reaction ratio of ~6:1 or higher facilitates the formation of a polynuclear complex. The mixed MeCN/PhCH_2_OH solvent mixture is necessary to ensure solubility of the reagents and to provide the alkoxido ligands. The metal centers are held together by nine 2.11 benzoato ligands, one 5.5-O^2−^ ion, four 3.3-O^2−^ ions and two 2.2-PhCH_2_O^−^ groups. The single terpy molecule is attached to the Mn^II^ center. The core topology (see Figure 4) is unique, comprising a {Mn^II^${\text{Mn}}_{3}^{\text{III}}$O_2_} butterfly and a {${\text{Mn}}_{3}^{\text{III}}$Mn^IV^O_4_} cubane sharing a Mn^III^ ion and the 5.5 (μ_5_) oxido group. The complex possesses an *S* = 6 ground state spin (*D* = −0.18 cm^−1^, *g* = 1.86). Single-crystal magnetization vs. dc field scans show only very weak hysteresis at 0.1 K.

(8) $$\begin{array}{l} 7\;({\text{Bu}}_{\;4}^{\text{n}}\text{N})\left[{\text{Mn}}_{4}^{\text{III}}{\text{O}}_{2}{\left({\text{O}}_{2}\text{CPh}\right)}_{9}\left({\text{H}}_{2}\text{O}\right)\right]+4\text{ terpy}\\ \;+8\;{\text{PhCH}}_{2}\text{OH}\overset{\text{MeCN}/{\text{PhCH}}_{2}\text{OH}}{\to }4[{\text{Mn}}^{\text{II}}{\text{Mn}}_{5}^{\text{III}}{\text{Mn}}^{\text{IV}}\;\\ \;{\text{O}}_{5}{\left({\text{OCH}}_{2}\text{Ph}\right)}_{2}{\left({\text{O}}_{2}\text{CPh}\right)}_{9}\left(\text{terpy}\right)]+7\;\left({\text{Bu}}_{\;4}^{\text{n}}\text{N}\right)\left({\text{O}}_{2}\text{CPh}\right)\\ \;+20\;{\text{PhCO}}_{2}\text{H}+{\text{H}}_{2}\text{O} \end{array}$$

### Phosphonates, a useful alternative to carboxylates

Phosphonates (${\text{RPO}}_{3}^{2-}$) are ideal ligands for stabilizing 3d-metal coordination clusters, some of which are SMMs. They possess three O donor atoms and can, in principle link to a maximum of nine metal centers. However, polymeric 3d-metal phosphonates are very insoluble and the isolation of phosphonato complexes is problematic. Three routes have been used to overcome the insolubility problem and stabilize discrete molecular species (Langley et al., 2008). First, solvothermal techniques can improve the solubilities. Second, a “co-ligand” can be used to maintain some solubility. And third, pre-formed carboxylato clusters can be linked in various ways by displacing some carboxylate groups by phosphonato ligands. For preparing 3d-metal phosphonate SMMs, there is one disadvantage and one advantage. The disadvantage is that phosphonato ligands propagate weak magnetic exchange interactions between metal centers, but the presence of bridging co-ligands (including monoatomic ones) can improve the situation. The advantage is that most metal phosphonates form 2D lattices; if polymerization can be stopped, e.g., by using other ligands, this would be ideal for creating structural anisotropy which, in turn, can lead to the desirable magnetic anisotropy.

Compound (Et_3_NH)[${\text{Co}}_{8}^{\text{II}}$(NO_3_)_3_(O_3_PPh)_2_(chp)_10_(Hchp)_2_] (**11**), where Hchp is 6-chloro-2-hydroxypyridine (**A** with R = Cl in Figure 1) and ${\text{PhPO}}_{3}^{2-}$ is the dianion of phenylphosphonic acid, is prepared by the reaction of Co(NO_3_)_2_·6H_2_O, Hchp and PhPO_3_H_2_ in MeCN in the presence of Et_3_N, Equation (9); the yield is ~50%. To retain the reagents in solution, the phenylphosphonic acid: Co(II) ratio should be low and Hchp has to be added before PhPO_3_H_2_. The base is necessary to deprotonate the two ligands. The two P atoms and the four Co^II^ ions lie on the vertices of a trigonal prism. The remaining four metal ions lie above and below the triangular faces of the prism. The phosphonato ligands both adopt the 4.211 mode (**HH** in Figure 2), while eight of the chp^−^ ligands bind with the 2.21 mode (**II** in Figure 2) and two with the 3.21 mode (**JJ** in Figure 2). The two neutral Hchp molecules are terminally coordinated exhibiting the 1.10 mode. The structure also contains two chelating nitrato groups (1.110) and a bridging 2.110 nitrato group (Langley et al., 2008). Study of the dynamic behavior of the magnetization shows that **11** displays slow magnetization relaxation. It was found that different samples gave different *U*_eff_ values and a range of pre-exponential factors, τ_0_. For example, one sample gave *U*_eff_ = 58.4(±2) cm^−1^ and τ_0_ = 1.8(±3) × 10^−12^ s, and another gave *U*_eff_ = 55.6(±2) cm^−1^ and τ_0_ = 2.1 (±3) × 10^−11^s.

(9) $$\begin{array}{l} 8\;{\left(\text{Co}\right)}^{\text{II}}{\left({\text{NO}}_{\text{3}}\right)}_{2}\cdot 6{\text{H}}_{\text{2}}\text{O}+12\text{ Hchp}+2\;{\text{PhPO}}_{3}{\text{H}}_{2}\\ \;+14\;{\text{Et}}_{3}\text{N}\overset{\text{MeCN}}{\to }({\text{Et}}_{3}\text{NH})[{\text{Co}}_{8}^{\text{II}}{\left({\text{NO}}_{3}\right)}_{3}{\left({\text{O}}_{3}\text{PPh}\right)}_{2}{\left(\text{chp}\right)}_{10}\\ \;{\left(\text{Hchp}\right)}_{2}]+13\;\left({\text{Et}}_{3}\text{NH}\right)\left({\text{NO}}_{3}\right)+48\;{\text{H}}_{2}\text{O} \end{array}$$

The reaction between the pre-formed oxido-centered triangle [${\text{Mn}}_{3}^{\text{II},\text{III},\text{III}}$O(O_2_CPh)_6_(py)_2_(H_2_O)] and benzylphosphonic acid (PhCH_2_PO_3_H_2_) in MeOH/MeCN, in the presence of LiOMe, in an 1:1:1 ratio, gives complex [${\text{LiMn}}_{2}^{\text{II}}$${\text{Mn}}_{10}^{\text{III}}$O_4_(OMe)_4_(O_2_CPh)_15_(O_3_PCH_2_Ph)_4_(py)_2_] (**12**) in 60% yield, Equation (10). The core of the complex consists of a central trigonal prism. Two of the square faces of the prism are each bridged to one edge of a peripheral triangular unit by two 5.221 phosphonato ligands (**KK** in Figure 2) and two 2.2 methoxido groups. The Li^I^ atom sits in the cavity of the central trigonal prism and forms six bonds to oxygen atoms. The remaining edges of the peripheral triangular units are unchanged from those in the starting material, i.e., are bridged by 2.11 ${\text{PhCO}}_{2}^{-}$ groups. The oxido atom in the triangular Mn units are μ_3_ (3.3), again as found in the starting Mn triangle. Interestingly, the crystal lattice contains two different {${\text{Mn}}_{2}^{\text{II}}$${\text{Mn}}_{10}^{\text{III}}$} molecules which have the same connectivity, but which are Jahn-Teller isomers of one another. The complex has a spin ground state of *S* = 6, but the small anisotropy of the system makes the compound a poor SMM with a very low *U*_eff_ (Shanmugam et al., 2006).

(10) $$\begin{array}{l} 4\;[{\text{Mn}}_{3}^{\text{II},\text{III},\text{III}}\text{O}{\left({\text{O}}_{\text{2}}\text{CPh}\right)}_{6}{\left(\text{py}\right)}_{2}\left({\text{H}}_{\text{2}}\text{O}\right)]+4\;{\text{PhCH}}_{2}{\text{PO}}_{3}{\text{H}}_{2}\\ \;+4\text{ LiOMe}+1/2\;{\text{O}}_{2}\;\overset{\text{MeOH}/\text{MeCN}}{\to }\;[{\text{LiMn}}_{2}^{\text{II}}{\text{Mn}}_{10}^{\text{III}}{\text{O}}_{4}{\left(\text{OMe}\right)}_{4}\\ \;{\left({\text{O}}_{2}\text{CPh}\right)}_{15}{\left({\text{O}}_{3}{\text{PCH}}_{2}\text{Ph}\right)}_{4}{\left(\text{py}\right)}_{2}]+3\text{ Li}\left({\text{O}}_{2}\text{CPh}\right)+6\;\left(\text{pyH}\right)\\ \;\left({\text{O}}_{2}\text{CPh}\right)+5\;{\text{H}}_{2}\text{O}+6\text{ py} \end{array}$$

### “Dancing” with azides

The azide (${\text{N}}_{3}^{-}$) ion is a very popular inorganic ligand in transition-metal cluster chemistry (Escuer and Aromi, 2006; Stamatatos and Christou, 2009; Escuer et al., 2014). In addition to its terminal coordination mode (1.10), it often participates as a bridging ligand in transition-metal chemistry. No less than 8 bridging modes have to-date been crystallographically characterized for the azido ligand. These are end-to-end (EE) (syn, syn-2.11, and syn, anti-2.11), end-on (EO) (2.20, 3.30, 4.40) and mixed EE/EO (3.21, 4.22, 6.33) modes. The bridging azido group is an effective magnetic coupler and it is thus not surprising that it is an ingredient in many 3d-metal SMMs. EE azido groups generally promote antiferromagnetic exchange interactions, whereas the EO ligation mode is responsible for ferromagnetic exchange interactions; however, there are exceptions to this general rule. There have been reported many reactions schemes that lead to azido-bridged 3d-metal coordination clusters and SMMs. These include, among others, systems intended to provide azides as the only bridging ligands in the cluster, primary organic ligand/azide “blends,” and reaction mixtures containing two different organic ligands and ${\text{N}}_{3}^{-}$ ions. In the following, we give a representative example of SMMs from each reaction system.

**Figure 5:**
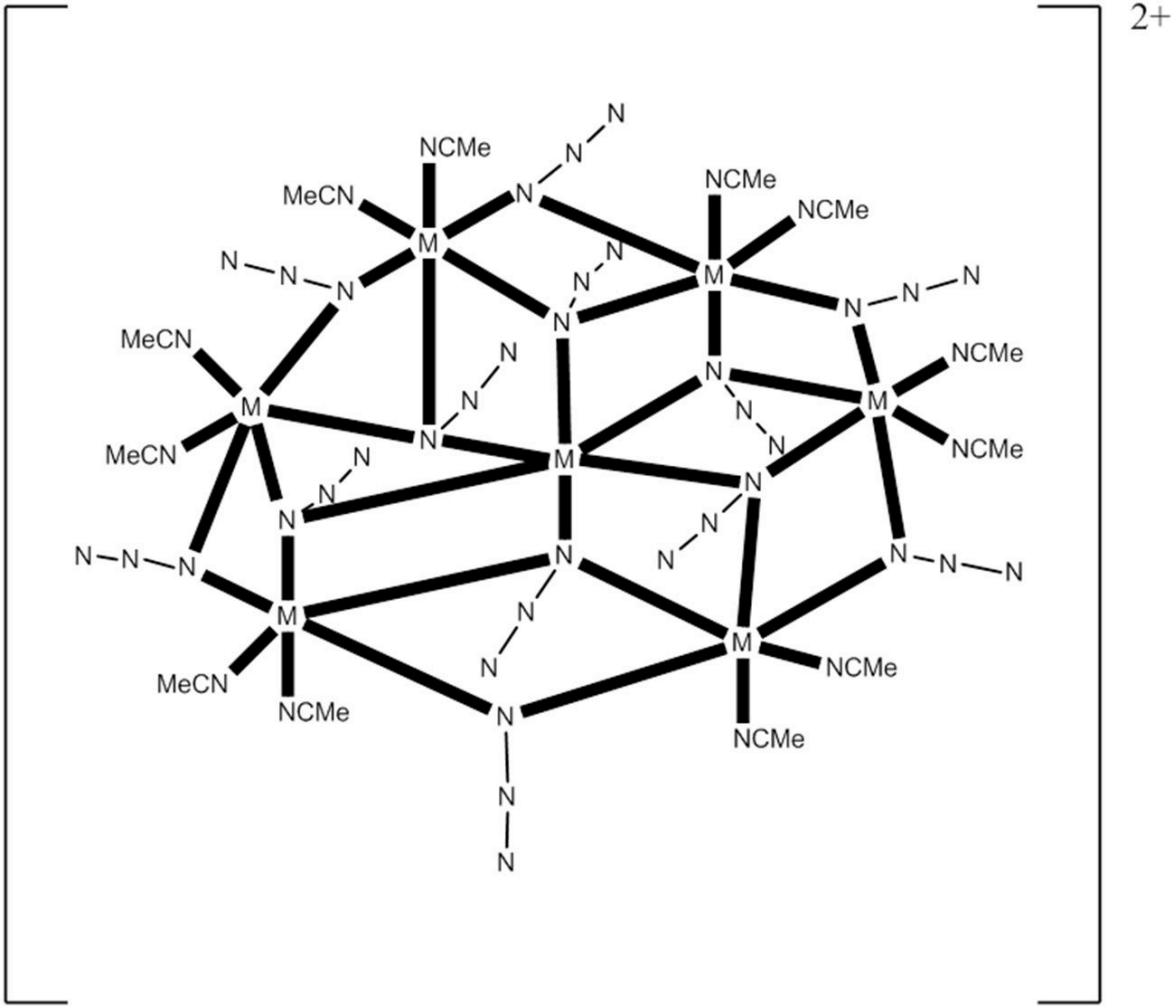
Schematic molecular structure of the heptanuclear cations of complexes **13** and **14**; coordination bonds are drawn in bold (Schematic drawing inspired by Alexandropoulos et al., 2014).

The 1:1:4 reaction between Co(ClO_4_)_2_·6H_2_O, Et_3_N, and Me_3_SiN_3_ in MeCN gives a pink solution which, upon layering with Et_2_O, leads to the heptanuclear cluster [${\text{Co}}_{7}^{\text{II}}$(N_3_)_12_(MeCN)_12_](ClO_4_)_2_ (**13**), Equation (11), in ~75% yield. Replacement of Co(ClO_4_)_2_·6H_2_O with Ni(ClO_4_)_2_·6H_2_O in the reaction gives the isomorphous compound [${\text{Ni}}_{7}^{\text{II}}$(N_3_)_12_(MeCN)_12_](ClO_4_)_2_ (**14**) (Alexandropoulos et al., 2014). The presence of Et_3_N is necessary for the isolation of the cluster, but its role appears unclear. The heptanuclear cations of **13** and **14** consist of an hexagon of M^II^ atoms (M = Co, Ni) surrounding a seventh metal ion in the center of the hexagon. The disk-like core is completed by 12 N atoms of six 3.30 and six 2.20 EO ${\text{N}}_{3}^{-}$ groups. The 3.30 azido groups bridge the ${\text{M}}_{6}^{\text{II}}$ hexagon with the central metal ion, while the 2.20 ones bridge the ${\text{M}}_{2}^{\text{II}}$ pairs of the hexagon. Two MeCN molecules are coordinated to each external metal center (see Figure 5). The cations have a layered structure, with layers of azido N atoms above and below the ${\text{M}}_{7}^{\text{II}}$ plane. There are strong intracluster ferromagnetic Co^II^…Co^II^ interactions in **13**, which leads to an effective spin ground state of *S* = 7/2; the complex is SMM under an external dc field of 0.1 T (*U*_eff_ = ~20 cm^−1^, τ_0_ = 8.0 × 10^−8^ s). The cluster cation has a very well-isolated ground-state spin value as indicated by the similar EPR spectra at 4 and 23 K.

(11) $$\begin{array}{l} 7\;{\text{Co}}^{\text{II}}{\left({\text{ClO}}_{4}\right)}_{2}\cdot \;6\;{\text{H}}_{2}\text{O}+12\;{\text{Me}}_{3}{\text{SiN}}_{3}+12\text{ MeCN}\overset{\text{MeCN}}{\underset{{\text{Et}}_{3}\text{N}}{\to }}\\ \;[{\text{Co}}_{7}^{\text{II}}{\left({\text{N}}_{3}\right)}_{12}{\left(\text{MeCN}\right)}_{12}]{\left({\text{ClO}}_{4}\right)}_{2}+12\;{\text{Me}}_{3}\text{Si}\left({\text{ClO}}_{4}\right)+42\;{\text{H}}_{2}\text{O} \end{array}$$

The ligand “blend” sao^2−^/${\text{N}}_{3}^{-,}$ where sao^2−^ is the dianion of salicylaldoxime (saoH_2_; **L** with R = H in Figure 1), in Mn chemistry has provided access to a structurally interesting SMM (Yang et al., 2007). The reaction of MnCl_2_·6H_2_O, NaN_3_, saoH_2_, and (Et_4_N)OH in MeOH gives a dark green solution from which (Et_4_N)_3_[${\text{Mn}}_{2}^{\text{II}}$${\text{Mn}}_{3}^{\text{III}}$O(N_3_)_6_Cl_2_(sao)_3_] (**15**) can be crystallized in low yield (~15%), Equation (12). The five metal ions define a trigonal bipyramid in which the two tetrahedral Mn^II^ atoms occupy the axial positions and the distorted octahedral Mn^III^ atoms are in the equatorial plane linked by a 3.3-O^2−^ group. Each Mn^II^ atom is connected to the equatorial Mn^III^ centers with three 2.20 azido groups, while a terminal chlorido group completes four-coordination at each Mn^II^. The Mn^III^ atoms are bridged by three 2.111 sao^2−^ ligands (**LL** with M = Mn^III^ and R = H in Figure 2). The anion has a ground-state spin of *S* = 11, with a first excited state *S* = 10 being 3.2 cm^−1^ higher in energy. Fit of the magnetization data gave a *D* value of−0.22 cm^−1^. The cluster is SMM with a *U*_eff_ value of 28 cm^−1^.

(12) $$\begin{array}{l} 5\;{\text{Mn}}^{\text{II}}{\text{Cl}}_{2}\cdot 6\;{\text{H}}_{2}\text{O}+6\;{\text{NaN}}_{3}+3\;{\text{saoH}}_{2}+5\;\left({\text{Et}}_{4}\text{N}\right)\text{OH}\\ \;+3/4\;{\text{O}}_{2}\overset{\text{MeOH}}{\to }{\left({\text{Et}}_{4}\text{N}\right)}_{3}[{\text{Mn}}_{2}^{\text{II}}{\text{Mn}}_{3}^{\text{III}}\text{O}{\left({\text{N}}_{3}\right)}_{6}{\text{Cl}}_{2}{\left(\text{sao}\right)}_{3}]\\ \;+6\text{ NaCl}+2\;\left({\text{Et}}_{4}\text{N}\right)\text{Cl}+51/2\;{\text{H}}_{2}\text{O} \end{array}$$

**Figure 6:**
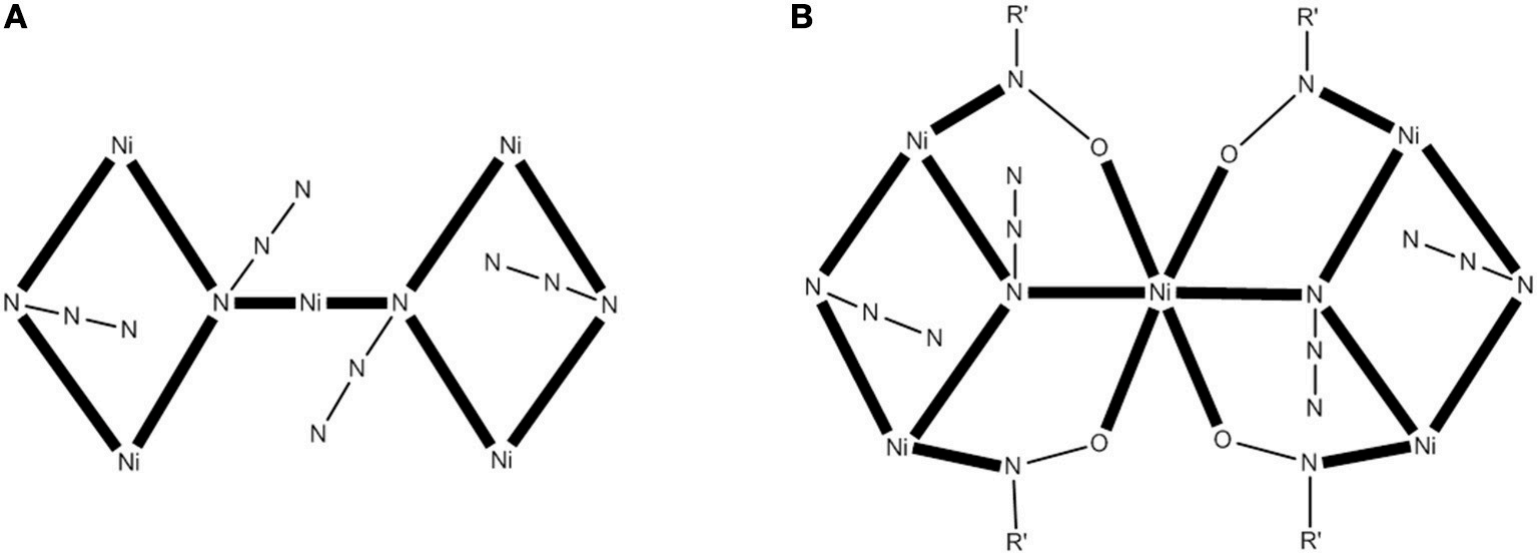
The {Ni_5_(2.20-N_3_)_2_ (3.30-N_3_)_2_}^6+^ core of the complex **16 (A)** and a more detailed representation of the core **(B)** emphasizing its {Ni_5_(2.20-N_3_)_2_ (3.30-N_3_)_2_ (2.11-0NR')_4_}^2+^ description (R′NO^−^ = ppko^−^); coordination bonds are drawn in bold (Schematic drawing inspired by Papatriantafyllopoulou et al., 2010).

A modern trend in 3d-metal azide cluster chemistry is the employment of two organic ligands (a primary and an ancillary) and ${\text{N}}_{3}^{-}$ ions in the reaction systems. The loss of a degree of synthetic control seems to be, at first glance, disadvantageous; however, this loss is more than compensated for by the large diversity of structural types expected. We present an example in which the ancillary organic ligand is a carboxylate ion. The reaction of Ni(ClO_4_)_2_6H_2_O, phenyl 2-pyridyl ketoxime (ppkoH; **H** with R = Ph in Figure 1), NaN_3_ and NaO_2_CH in a ~1:1:1:1.5 ratio in MeOH gives a red-brown solution, from which can be subsequently isolated complex [Ni_5_(N_3_)_4_(O_2_CH)_2_(ppko)_4_(MeOH)_4_] (**16**) in ~50% yield, Equation (13). The structurally novel {Ni_5_(2.20-N_3_)_2_(3.30-N_3_)_2_}^6+^ core (see Figure 6A) contains the five metal ions in a bowtie topology (two Ni_3_ triangles with a common vertex). Each diatomic oximate group of a 2.111 ppko^−^ (**MM** with R = Ph and M = Ni in Figure 2) bridges the central Ni^II^ atom and a peripheral one (see Figure 6B), while a 2.11 formato ligand spans the base of each isosceles triangle. Magnetic data indicate an overall ferromagnetic behavior (*S* = 5 ground state). Ac magnetic susceptibility studies reveal non-zero, frequency-dependent out-of-phase ($\chi_{\text{M}}^{\text{“}}$) signals at temperatures below ~3.5 K. However, single-crystal magnetization vs. dc field scans at variable temperatures and variable sweep rates down to 0.04 K show no significant hysteresis loops, except minor ones at 0.04 K that are assigned to weak intermolecular interactions (Papatriantafyllopoulou et al., 2010).

(13) $$\begin{array}{l} 5\text{ Ni}{\left({\text{ClO}}_{4}\right)}_{2}\cdot 6{\text{H}}_{2}\text{O}+4\;{\text{NaN}}_{3}+6\;{\text{NaO}}_{2}\text{CH}+4\text{ ppkoH}\\ \;+4\text{ MeOH}\overset{\text{MeOH}}{\to }[{\text{Ni}}_{5}{\left({\text{N}}_{3}\right)}_{4}{\left({\text{O}}_{2}\text{CH}\right)}_{2}{\left(\text{ppko}\right)}_{4}{\left(\text{MeOH}\right)}_{4}]\\ \;+4\;{\text{HCO}}_{2}+10\;{\text{NaClO}}_{4}+30\;{\text{H}}_{2}\text{O} \end{array}$$

### Comproportionation reactions in manganese SMM chemistry

A commonly used synthetic route for the preparation of high-oxidation state Mn clusters and SMMs is the comproportionation reaction of a Mn^II^ source and Mn^VII^${\text{O}}_{4}^{-}$ (i.e., Mn^VII^) in the presence of simple carboxylate ions and/or chelating/bridging ligands. Such reactions are very useful because they normally give products containing some Mn(IV), thus preventing complication from low-lying excited states which is a common situation with Mn^II^…Mn^III^ and Mn^III^…Mn^III^ couplings. This was the original method for the synthesis of the {${\text{Mn}}_{8}^{\text{III}}$${\text{Mn}}_{4}^{\text{IV}}$} complex **1** which, in a sense, initiated the area of SMMs (Lis, 1980). The reaction of Mn^II^(O_2_CMe)·4H_2_O and KMnO_4_ in 60% v/v MeCO_2_H/H_2_O, in a ratio appropriate for the final +3.33 Mn oxidation level and under warm heating, provides access to high yields of crystalline **1**·2H_2_O·4MeCO_2_H, Equation (14), in the form of reddish-black crystals. The crystals were of X-ray quality and the structure of the cluster was solved by single-crystal, X-ray crystallography.

(14) $$\begin{array}{l} 44\;{\text{Mn}}^{\text{II}}+16\;{\text{Mn}}^{\text{VII}}\;\to \;5\;\{{\text{Mn}}_{8}^{\text{III}}{\text{Mn}}_{4}^{\text{IV}}\} \end{array}$$

The prototype SMM **1** can also be isolated in high yields by the oxidation of [${\text{Mn}}_{3}^{\text{III}}$O(O_2_CMe)_6_(py)_3_](ClO_4_) (Vincent et al., 1987) by KMnO_4_ in 60% v/v MeCO_2_/H_2_O in a ratio designed to **1** (Sessoli et al., 1993b), Equation (15).

(15) $$\begin{array}{l} 0.29\;{\text{Mn}}^{7+}+1.15\;{\text{Mn}}_{3}^{3+\;}\;\to \;3.72\;{\text{Mn}}^{3.33+} \end{array}$$

An ideal high-oxidation Mn(VII) reagent for such reactions in organic solvents has been documented to be (Bu^*n*^_4_N)(MnO_4_) (Vincent et al., 1986); this oxidizing reagent is soluble in many organic solvents. It is easily prepared by mixing aqueous solutions of KMnO_4_ and (Bu^*n*^_4_N)Br in slight excess; it is pure enough for use without recrystallization and storage at −5°C increases its stability (storage at room temperature leads to slow decomposition in a period of ~1 month give a brown sticky material). The benzoato analog of **1**, i.e., [${\text{Mn}}_{8}^{\text{III}}$${\text{Mn}}_{4}^{\text{IV}}$O_12_(O_2_CPh)_16_(H_2_O)] (**17**), also a good SMM, can be prepared in low yield (~10%) by comproportionation of Mn(O_2_CMe)4H_2_O with (Bu^*n*^_4_N)(MnO_4_) in the presence of PhCO_2_H in py/MeCN (Sessoli et al., 1993b). Although the Mn^II^:${\text{MnO}}_{4}^{-}$ ratio was identical to that in Equation (14), the major product of this reaction is [${\text{Mn}}_{3}^{\text{II},\text{III},\text{III}}$O(O_2_CPh)_6_(py)_2_(H_2_O)], i.e., in this case only a small amount of higher (>III) average oxidation product is obtained.

An interesting comproportionation reaction to manganese (III/IV) pivalato clusters gave a half-integer spin SMM (Mukherjee et al., 2013). The 1:1:1:1 comproportionation reaction of Mn^II^(O_2_CBu^*t*^)_2_·2H_2_O, Mn^II^(ClO_4_)_2_·6H_2_O, (Bu^*n*^_4_N)Cl and (Bu^*n*^_4_N)(MnO_4_) in the presence of a large excess of Bu^*t*^CO_2_H (pivalic acid; its excess is necessary to prevent precipitation of amorphous Mn oxides) in hot MeCN gives a dark brown solution that leads to the isolation of [${\text{Mn}}_{7}^{\text{III}}$Mn^IV^O_6_(OH)Cl_3_(O_2_CBu^*t*^)_9_(Bu^*t*^CO_2_H)_0.5_(MeCN)_0.5_] (**18**) in ~25% yield. The average metal oxidation state in the reaction is +4.5, Equation (16), but in the product it is less (~+3.1). The low yield of **18** is an evidence that there are probably other higher oxidation-state products in the mother liquor which are not precipitated. Use of MnCl_2_ or Mn(NO_3_)_2_, instead of the manganese(II) perchlorate salt, also give **18**, but in lower yields. It was seemed possible that the low Mn(IV) content in **18** is due to the fact that ${\text{MnO}}_{4}^{-}$ oxidizes not only Mn(II) but also the solvent. The use of an even lower Mn(II):Mn(VII) reaction ratio of 2:3 to increase the average oxidation state to +5, Equation (17), gives a mixture of **18** and [${\text{Mn}}_{2}^{\text{III}}$${\text{Mn}}_{6}^{\text{IV}}$O_9_(O_2_CBu^*t*^)_12_] (**19**); the latter can be prepared pure from a simplified comproportionation reaction between Mn(NO_3_)_2_ and (Bu^*n*^_4_N)(MnO_4_) (2:3) in the presence of pivalic acid in hot MeCN in ~35% yield. The average metal oxidation state in **19** is now +3.75.

(16) $$\begin{array}{l} {\text{Mn}}^{2+}+{\text{Mn}}^{7+\;}\;\to \;2\;{\text{Mn}}^{4.5+}\; \end{array}$$

(17) $$\begin{array}{l} \;2\;{\text{Mn}}^{2+}\;+3\;{\text{Mn}}^{7+}\;\to 2\;{\text{Mn}}^{5+} \end{array}$$

Complex **18** possesses a {${\text{Mn}}_{7}^{\text{III}}$Mn^IV^(3.3-O)_4_(4.4-O)_2_(3.3-OH)(4.4-Cl)(2.2-Cl)}^10+^ core containing two body-fused Mn_4_ butterfly subunits attached to the remaining Mn ions through bridging oxido, hydroxide, and chlorido groups. Cluster **19** possesses a {${\text{Mn}}_{2}^{\text{III}}$${\text{Mn}}_{6}^{\text{IV}}$(3.3-O)_6_(2.2-O)_3_}^12+^ core consisting of two incomplete {${\text{Mn}}_{3}^{\text{IV}}$O_4_} cubanes linked to two Mn^III^ atoms. Solid-state dc and ac magnetic susceptibility data have indicated an *S* = 15/2 ground state for **18**; the *D* value is −0.22(2) cm^−1^. The ac susceptibility data revealed non-zero, frequency-dependent χ”_M_ signals for this complex below 3 K, providing a strong evidence for SMM behavior; this behavior was confirmed by the appearance of hysteresis loops in single-crystal magnetization vs. dc field studies. The loops display two well-resolved QTM steps. The complex is a half-integer cluster and it is not expected to display QTM in the absence of transverse fields; the latter are provided by the transverse components of dipolar and exchange fields from neighboring molecules, and hyperfine fields from ^55^Mn (*I* = 15/2) nuclei. The relaxation rate at zero applied field is very fast at several temperatures, and this prevented the determination of *U*_eff_. Complex **19** does not exhibit χ“_M_ signals above 1.8 K.

### The reductive aggregation route and its modifications in manganese SMM chemistry

The reductive aggregation route in Mn chemistry represents a modification of the comproportionation procedure. The Mn(II) source is omitted and MeOH is used not only as a solvent, but also both as the reducing agent for Mn(IV) and as a potential source of bridging MeO^−^ groups, in the presence of an excess of carboxylic acid that prevents precipitates of Mn hydroxides and/or oxides, and provides carboxylato ligands.

The reaction of phenylacetic acid (PhCH_2_CO_2_H) with (Bu^*n*^_4_N)(MnO_4_) in MeOH leads to a dark brown solution and isolation of [${\text{Mn}}_{10}^{\text{III}}$${\text{Mn}}_{6}^{\text{IV}}$O_16_(OMe)_6_(O_2_CCH_2_Ph)_16_(MeOH)_6_] (**20**) (King et al., 2004), eqn. (18). In Equation (18), it is assumed that MeOH is oxidized to formaldehyde. The yield is low (~10%), but the reaction is perfectly reproducible and gives crystalline material. Such reactions are certainly complicated with several cluster species in solution; the final outcome depends on several factors, such as relative solubilities, kinetics of crystallization and lattice energies. An important issue of this reaction (and several reactions of this type) is the necessity of using small volumes of MeOH; dilute solutions lead to Mn hydroxides and/or oxides. In the reaction that leads to **20**, use of EtOH instead of MeOH leads also to hydroxides/oxides, whereas 2-propanol leads to pale yellow solutions indicative of full reduction of Mn(VII) to Mn(II). The metal centers are linked through 14 3.3-O^2−^, 2.2-O^2−^, 4 2.2-MeO^−^, and 2 2.11-O_2_CCH_2_Ph ligands, the remaining 14 2.11-O_2_CCH_2_Ph^−^, 2 2.2-MeO^−^, and 6 1.1-MeOH ligands providing peripheral ligation. A useful description of the complex is as a very small piece of a Mn oxide mineral held within a nonplanar ring of Mn^III^ atoms. There are dominant antiferromagnetic exchange interactions in the molecule. Low-lying states make a valid assignment for the total ground-state spin of **20** difficult; it is most probably *S* = 2. Hysteresis behavior is clearly visible below 1 K, indicating SMM properties.

(18) $$\begin{array}{l} 16\;\left({\text{Bu}}^{\text{n}}{}_{4}\text{N}\right)\left({\text{Mn}}^{\text{VII}}{\text{O}}_{4}\right)+32\;{\text{PhCH}}_{2}{\text{CO}}_{2}\text{H}+41\;{\text{CH}}_{3}\text{OH}\overset{\text{MeOH}}{\to }\\ \;\left[{\text{Mn}}_{10}^{\text{III}}{\text{Mn}}_{6}^{\text{IV}}{\text{O}}_{16}{\left({\text{OCH}}_{3}\right)}_{6}{\left({\text{O}}_{2}{\text{CCH}}_{2}\text{Ph}\right)}_{16}{\left({\text{CH}}_{3}\text{OH}\right)}_{6}\right]\\ \;+29\text{ HCHO}+16\;\left({\text{Bu}}^{\text{n}}{}_{4}\text{N}\right)\left({\text{O}}_{2}{\text{CCH}}_{2}\text{Ph}\right)+48\;{\text{H}}_{2}\text{O} \end{array}$$

An analogous reaction, but using Bu^*n*^CO_2_H this time, leads to the low-yield preparation of [${\text{Mn}}_{8}^{\text{III}}$${\text{Mn}}_{4}^{\text{IV}}$O_10_(OMe)_4_(O_2_CBu^*t*^)_16_(MeOH)_2_] (**21**), Equation (19). The 12 Mn centers are held together by 10 3.3-O^2−^ and 4 2.2-MeO^−^ groups. The molecule possesses a central face-fused defective dicubane core of four Mn^IV^ atoms held within a non-planar ring of eight Mn^III^ atoms to give an overall chair conformation. The complex has an *S* = 9 ground state and is SMM (King et al., 2005). No sign of any steps was observed in the hysteresis loops as a result of broadening effects due to extensive disorder of most Bu^*t*^ groups. The reductive aggregation route has also led to anionic {${\text{Mn}}_{8}^{\text{III}}$${\text{Mn}}_{4}^{\text{IV}}$}^2−^ SMMs (Tasiopoulos et al., 2005).

(19) $$\begin{array}{l} 12\;\left({\text{Bu}}^{\text{n}}{}_{4}\text{N}\right)\left({\text{Mn}}^{\text{VII}}{\text{O}}_{4}\right)+28\;{\text{Bu}}^{\text{t}}{\text{CO}}_{2}\text{H}+28\;{\text{CH}}_{3}\text{OH}\overset{\text{MeOH}}{\to }\\ \;\left[{\text{Mn}}_{8}^{\text{III}}{\text{Mn}}_{4}^{\text{IV}}{\text{O}}_{10}{\left({\text{OCH}}_{3}\right)}_{4}{\left({\text{O}}_{2}{\text{CBu}}^{\text{t}}\right)}_{16}{\left({\text{CH}}_{3}\text{OH}\right)}_{2}\right]\\ \;+22\text{ HCHO}+12\;\left({\text{Bu}}^{\text{n}}{}_{4}\text{N}\right)\left({\text{O}}_{2}{\text{CBu}}^{\text{t}}\right)+38\;{\text{H}}_{2}\text{O} \end{array}$$

A modification of the reductive aggregation route involves employment of a second (in addition to Mn^VII^${\text{O}}_{4}^{-}$) high oxidation-state Mn cluster. Thus, the [${\text{Mn}}_{8}^{\text{III}}$${\text{Mn}}_{4}^{\text{IV}}$O_12_(O_2_CMe)_16_(H_2_O)_4_] (**1**)/(Bu^*n*^_4_N)(Mn^VII^O_4_)/MeCO_2_H reaction mixtures in MeOH and EtOH lead to the giant torus-like SMMs [${\text{Mn}}_{84}^{\text{III}}$O_72_(OH)_6_(OMe)_24_(O_2_CMe)_78_(H_2_O)_42_(MeOH)_12_] (**22**) (Tasiopoulos et al., 2004) and [${\text{Mn}}_{70}^{\text{III}}$O_60_(OEt)_20_(O_2_CMe)_70_(H_2_O)_22_(EtOH)_16_] (**23**) (Vinslava et al., 2016), respectively, Equations (20) and (21). In these chemical equations, it is assumed that MeOH and EtOH are oxidized to formaldehyde and acetaldehyde, respectively. The *U*_eff_ value of the {${\text{Mn}}_{70}^{\text{III}}$} cluster is ~16 cm^−1^.

(20) $$\begin{array}{l} 6\;[{\text{Mn}}_{8}^{\text{III}}{\text{Mn}}_{4}^{\text{IV}}{\text{O}}_{12}{\left({\text{O}}_{2}\text{CMe}\right)}_{16}{\left({\text{H}}_{2}\text{O}\right)}_{4}]\\ \;+12\;\left({\text{Bu}}^{\text{n}}{}_{4}\text{N}\right)\left({\text{Mn}}^{\text{VII}}O_{4}\right)+72\;{\text{CH}}_{3}\text{OH}\overset{\text{MeOH}}{\underset{{\text{MeCO}}_{2}\text{H}}{\to }}\\ \left[{\text{Mn}}_{84}^{\text{III}}{\text{O}}_{72}{\left(\text{OH}\right)}_{6}{\left({\text{OCH}}_{3}\right)}_{24}{\left({\text{O}}_{2}\text{CMe}\right)}_{78}{\left({\text{H}}_{2}\text{O}\right)}_{42}{\left({\text{CH}}_{3}\text{OH}\right)}_{12}\right]\\ \;+12\;\left({\text{Bu}}^{\text{n}}{}_{4}\text{N}\right)\left({\text{O}}_{2}\text{CMe}\right)+36\text{ HCHO}+6\;{\text{MeCO}}_{2}\text{H}+24\;{\text{H}}_{2}\text{O} \end{array}$$

(21) $$\begin{array}{l} 5\;\left[{\text{Mn}}_{8}^{\text{III}}{\text{Mn}}_{4}^{\text{IV}}{\text{O}}_{12}{\left({\text{O}}_{2}\text{CMe}\right)}_{16}{\left({\text{H}}_{2}\text{O}\right)}_{4}\right]+10\;\left({\text{Bu}}^{\text{n}}{}_{4}\text{N}\right)\left({\text{Mn}}^{\text{VII}}{\text{O}}_{4}\right)\\ \;+66\;{\text{C}}_{2}{\text{H}}_{5}\text{OH}\overset{\text{EtOH}}{\underset{{\text{MeCO}}_{2}\text{H}}{\to }}[{\text{Mn}}_{70}^{\text{III}}{\text{O}}_{60}{\left({\text{OC}}_{2}{\text{H}}_{5}\right)}_{20}{\left({\text{O}}_{2}\text{CMe}\right)}_{70}\\ {\left({\text{H}}_{2}\text{O}\right)}_{22}{\left({\text{C}}_{2}{\text{H}}_{5}\text{OH}\right)}_{16}]+10\;\left({\text{Bu}}^{\text{n}}{}_{4}\text{N}\right)\left({\text{O}}_{2}\text{CMe}\right)+30\;{\text{CH}}_{3}\text{CHO}\\ \;+38\;{\text{H}}_{2}\text{O} \end{array}$$

### Procedures involving two different primary organic ligands

The combined use of two primary organic ligands with different functionalities in the reaction systems is another route for the construction of clusters and SMMs. This method has developed mainly by the groups of Tasiopoulos and Brechin. In the most of the reported examples, one of the primary organic ligands does not appear in the final product, but its role in solution appears essential for the reaction. This route has led, among others, to a giant {${\text{Mn}}_{18}^{\text{II}}$${\text{Mn}}_{14}^{\text{III}}$} double-decker wheel with an *S* = 11 or 12 ground state and SMM behavior (*U*_eff_ = ~31 cm^−1^, τ_0_ = 3.5 × 10^−12^ s); the small τ_0_ value has been attributed to low-lying excited states and weak intermolecular interactions (Manoli et al., 2011). We give below a representative, simpler example to illustrate the method; the product however, lacks SMM behavior, even to temperatures as low as 30 mK.

**Figure 7:**
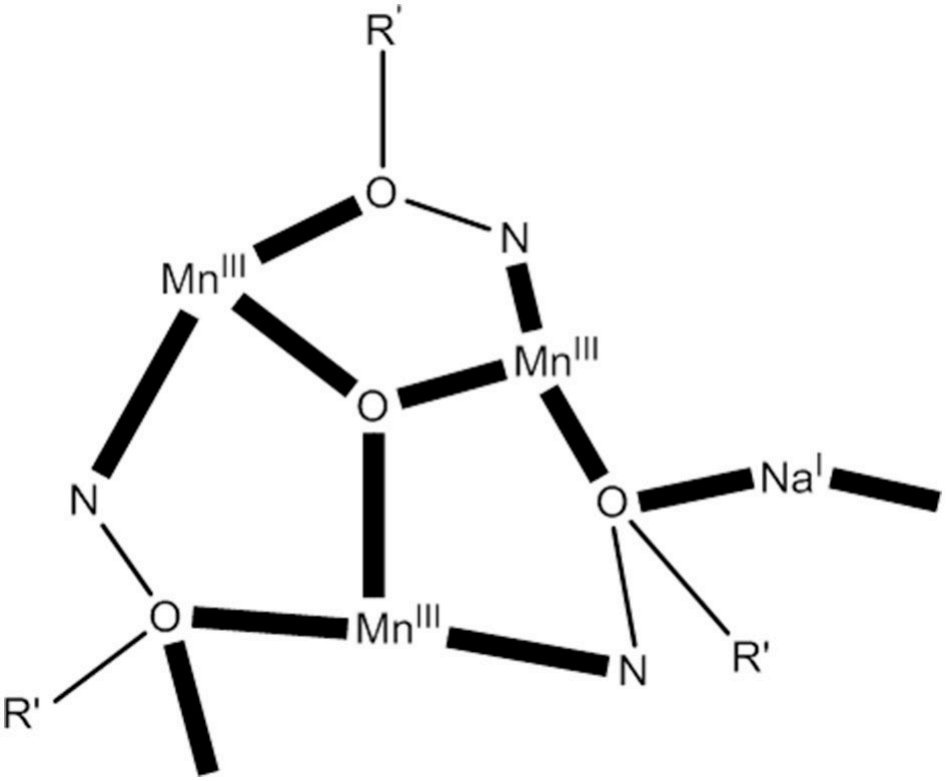
A simplified view of the {${\text{Mn}}_{3}^{\text{III}}$ Na^I^} repeating unit that forms the core of complex **24** (R′ NO = saph^2−^); coordination bonds are drawn in bold (Schematic drawing inspired by Manoli et al., 2016).

The 1:1:1:1 reaction of MnBr_2_·4H_2_O, 2-(hydroxymethyl)phenol (hpH_2_; **O** in Figure 1), saoH_2_ (**L** with R = H in Figure 1), and NaOCN in MeCN/DMF (4:1 v/v) leads to [${\text{Mn}}_{18}^{\text{III}}$${\text{Na}}_{6}^{\text{I}}$O_6_Br_12_(sao)_18_(H_2_O)_18_(DMF)_6_] (**24**) in typical yields of ~50% (Manoli et al., 2016), Equation (22). Reactions performed without the presence of the diol do not give this cluster, emphasizing its important role in the formation of **24**. The structure of **24** consists of a {${\text{Mn}}_{18}^{\text{III}}$${\text{Na}}_{6}^{\text{I}}$} wheel-like cluster comprising oximate-based {${\text{Mn}}_{3}^{\text{III}}$O}^7+^ triangular units linked through six Na^+^ ions. The {${\text{Mn}}_{3}^{\text{III}}$Na^I^} repeating unit possesses an oxo-centered triangular arrangement of Mn^III^ atoms and a Na^+^ ion attached to it via oxygen atoms (see Figure 7) of oximate groups which occupy the edges of the triangle. Two 3.211 (**NN** in Figure 2) and one 4.221 (**OO** in Figure 2) sao^2−^ ligands, as well as one 2.2 H_2_O molecule connect each Mn(III) triangle with two neighboring Na^+^ ions, which in turn are linked to the next triangular subunits forming the wheel.

(22) $$\begin{array}{l} 18\;{\text{Mn}}^{\text{II}}{\text{Br}}_{2}\cdot 4{\text{H}}_{2}\text{O}+30\text{ NaOCN}+18\;{\text{saoH}}_{2}+6\text{ DMF}\\ \;+9/2\;{\text{O}}_{2}\;\overset{\text{MeCN}/\text{DMF}}{\underset{{\text{hpH}}_{2}}{\to }}\;[{\text{Mn}}_{18}^{\text{III}}{\text{Na}}_{6}^{\text{I}}{\text{O}}_{6}{\text{Br}}_{12}{\left(\text{sao}\right)}_{18}{\left({\text{H}}_{2}\text{O}\right)}_{18}\\ \;{\left(\text{DMF}\right)}_{6}]+24\text{ NaBr}+30\text{ HOCN}+57\;{\text{H}}_{2}\text{O} \end{array}$$

## The years of design: strict and less strict synthetic strategies

All the routes described in section The Years of Innocence: “Try and See” Exercises depend on serendipity rather than on strict design; this means that the final product and its properties could not be predicted. However, those routes (and others not mentioned) were the basis of the great developments in the area of SMMs.

After ~2,000, scientists were more interested in designing the chemical and structural identity of the coordination clusters resulting from their efforts, and in predicting and controlling the magnetic properties of the products. Design principles and examples, which have been developed mainly in Mn SMM chemistry, will be presented in this chapter. The strategies are based on the modification of structural types already known that may or may not already be SMMs in their own right. From the structural viewpoint, two are the important parameters to be considered for such modifications: (a) the nuclearity of the coordination cluster, and (b) the core. Each of these parameters, or both, may change or may not change in the product with respect to the starting material (which is always a pre-formed cluster). From the magnetic viewpoint, the starting material may or may not be an SMM. In the former case, the primary goal of the synthetic strategy is to improve the SMM properties. In the latter, the main goal is to “switch on” SMM properties.

The choice of the below mentioned strategies is subjective and again we owe our apologies to many synthetic chemists whose elegant work will not be illustrated here.

### Substitution of carboxylato ligands by other carboxylato groups

Several groups (mainly Christou's group) have developed procedures to replace ${\text{RCO}}_{2}^{-}$ ligands in Mn SMMs with a variety of other R′${\text{CO}}_{2}^{-}$ groups. The replacement can be complete or partial (vide infra). The most studied SMM for such reactivity studies is **1**. As described in section Comproportionation Reactions in Manganese SMM Chemistry, complex **17** can be prepared using a comproportionation reaction, but in low yield (~10%). Treatment of **1** with 32 equivs of PhCO_2_H (100% excess) in CH_2_Cl_2_ leads to the exchange of most ${\text{MeCO}}_{2}^{-}$ ligands. For full replacement another ligand substitution cycle can be performed; the partially exchanged solid from the first cycle can be treated with other 32 equivs of PhCO_2_H giving the desired product in an overall >50% yield (Sessoli et al., 1993b), Equation (23).

(23) $$\begin{array}{l} \left[{\text{Mn}}_{8}^{\text{III}}{\text{Mn}}_{4}^{\text{IV}}{\text{O}}_{12}{\left({\text{O}}_{2}\text{CMe}\right)}_{16}{\left({\text{H}}_{2}\text{O}\right)}_{4}\right]\\ \;+16\;{\text{PhCO}}_{2}\text{H}\overset{\text{exc}{\text{.PhCO}}_{2}\text{H},{\text{CH}}_{2}{\text{Cl}}_{2}}{\underset{2\text{cycles}}{\to }}[{\text{Mn}}_{8}^{\text{III}}{\text{Mn}}_{4}^{\text{IV}}{\text{O}}_{12}{\left({\text{O}}_{2}\text{CPh}\right)}_{16}\\ \;{\left({\text{H}}_{2}\text{O}\right)}_{4}]+16\;{\text{MeCO}}_{2}\text{H} \end{array}$$

In general, such reactions are equilibria, Equation (24). The substitution reaction is facilitated by the higher acidity of the incoming carboxylic acid, R′CO_2_H, and its presence in excess. In the case of R = H (i.e., **1**), MeCO_2_H can be removed as its toluene azeotrope shifting the equilibrium represented by Equation (23) to the right. The distillation of the azeotrope of MeCO_2_H and toluene is a very valuable method for introducing R′${\text{CO}}_{2}^{-}$ groups whose conjugate acid has a p*K*_a_ value higher than that of MeCO_2_H or comparable to this (Bagai and Christou, 2009). The nuclearity and the core in the product are the same with those existing in the starting material. Such carboxylate substitution reactions often lead to better solubility in organic solvents and altered redox properties of the products compared to **1**.

(24) $$\begin{array}{l} \left[{\text{Mn}}_{8}^{\text{III}}{\text{Mn}}_{4}^{\text{IV}}{\text{O}}_{12}{\left({\text{O}}_{2}\text{CR}\right)}_{16}\;{\left({\text{H}}_{2}\text{O}\right)}_{4}\right]+16\;{\text{R}}^{\prime }{\text{CO}}_{2}\text{H}\\ \;⇌\left[{\text{Mn}}_{8}^{\text{III}}{\text{Mn}}_{4}^{\text{IV}}{\text{O}}_{12}{\left({\text{O}}_{2}\text{CR}\right)}_{\text{x}}{\left({\text{O}}_{2}{\text{CR}}^{\prime }\right)}_{16-\text{x}}\;{\left({\text{H}}_{2}\text{O}\right)}_{4}\right]\\ \;+\;\left(16-\text{x}\right)\;{\text{RCO}}_{2}\text{H}+\text{x }{\text{R}}^{\prime }{\text{CO}}_{2}\text{H} \end{array}$$

This type of carboxylate substitution is of important utility in several technological aspects of the chemistry of **1** and its derivatives (Domingo et al., 2012). We are doing a parenthesis here to mention that applications of SMMs require the development of strategies to move from bulk crystals to molecules that can be grafted on surfaces, sensors or other systems able to behave as a device; the challenge here is that the SMMs should retain their properties in the device. There are three routes for the nanostructuration of SMMs on surfaces from solution: (a) SMM direct deposition on a bare surface in order to immobilize the SMM through weak non-covalent interactions, (b) pre-functionalization of SMMs with groups that are able to interact chemically with the bare surface, and (c) pre-functionalization of the surface with appropriate groups that are able to interact with the SMM. An early example of pre-functionalization of SMMs for deposition onto an Au(111) surface involves replacement of the ${\text{MeCO}}_{2}^{-}$ groups of **1** by carboxylato ligands containing thiol groups (Cornia et al., 2003). The 16-sulfanylhexadecanoate (**P** in Figure 1) derivative of **1** was designed, in which the thiol groups were acetyl-protected (**Q** in Figure 1) to avoid undesirable reactivity in the presence of the Mn^III^ and Mn^IV^ centers. The *U*_eff_ value of the product is ~38 cm^−1^, close to that of **1**. The deposition was achieved by incubating the Au substrate in an SMM solution in a basic environment to ensure the deprotection of the thiol groups. The SMMs covered completely the surface with disordered layers, which could be disrupted by a continuous scanning of the area allowing the first imaging of discrete dodecanuclear Mn molecules by Scanning Tunneling Microscopy.

The carboxylate substitution can be partial and site-selective. In the case of the {${\text{Mn}}_{8}^{\text{III}}$${\text{Mn}}_{4}^{\text{IV}}$} SMMs, the products have the formula [${\text{Mn}}_{8}^{\text{III}}$${\text{Mn}}_{4}^{\text{IV}}$O_12_(O_2_CR)_8_(O_2_CR')_8_(H_2_O)_4_], i.e., x = 8 in Equation (24). The site-selective (or specific) substitution results from the different reactivity to electrophiles of some carboxylate ligands in **1**, **17** and related SMMs. Of the 16 carboxylates, 4 (type **I**) have both their O atoms on Jahn-Teller elongation axes of Mn^III^ atoms, 4 (type **I′**) have one, and 8 (type **I″**) have none being equatorial. Since the Mn^III^-O bonds on the Jahn-Teller axis are weaker than those on the axes of the equatorial plane, the relative susceptibility of the carboxylate groups to electrophilic attack would be expected to be **I**>**I′>** **I″** (Bagai and Christou, 2009) and this has been nicely demonstrated in several cases. Another relevant synthetic hypothesis is that the more basic carboxylate groups would thermodynamically prefer to bind at the non-Jahn-Teller equatorial sites (type **I″**) where they can form the strongest Mn^III^-O bonds.

Examples confirming these synthetic hypotheses are the reactions illustrated by Equations (25–28). Addition of 8 equivs of CHCl_2_CO_2_H (p*K*_a_ = 1.48) to [${\text{Mn}}_{8}^{\text{III}}$${\text{Mn}}_{4}^{\text{IV}}$O_12_(O_2_CCH_2_^*t*^Bu)_16_(H_2_O)_4_] (**25**) or [${\text{Mn}}_{8}^{\text{III}}$${\text{Mn}}_{4}^{\text{IV}}$O_12_(O_2_CEt)_16_(H_2_O)_3_] (**26**) in CH_2_Cl_2_ gives [${\text{Mn}}_{8}^{\text{III}}$${\text{Mn}}_{4}^{\text{IV}}$O_12_(O_2_CCHCl_2_)_8_(O_2_CCH_2_^*t*^Bu)_8_(H_2_O)_3_] (**27**) or [${\text{Mn}}_{8}^{\text{III}}$${\text{Mn}}_{4}^{\text{IV}}$O_12_(O_2_CCHCl_2_)_8_(O_2_CEt)_8_(H_2_O)_3_] (**28**), respectively; the p*K*_a_ values of Bu^*t*^CH_2_COOH and EtCO_2_H are 5.24 and 4.86, respectively, and the order of basicities is thus Bu^*t*^CH_2_${\text{CO}}_{2}^{-}$ > ${\text{EtCO}}_{2}^{-}$ >> CHCl_2_${\text{CO}}_{2}^{-}$ (Soler et al., 2001). In the structures of both **27** and **28**, the CHCl_2_${\text{CO}}_{2}^{-}$ groups are in axial sites and the pre-existed ${\text{RCO}}_{2}^{-}$ groups (R = Bu^*t*^CH_2_, Et) are equatorial. Thus, the CHCl_2_${\text{CO}}_{2}^{-}$ ligands are either bridging Mn^III^/Mn^III^ pairs with both their O atoms lying on the Jahn-Teller axes, or they are bridging Mn^III^/Mn^IV^ pairs with only one O atom on a Mn^III^ Jahn-Teller elongation axis. The 8 equatorial, more basic ${\text{RCO}}_{2}^{-}$ ligands bridge Mn^III^/Mn^III^ pairs and there are no O atoms on the Jahn-Teller axes. Complexes **27** and **28** can also be prepared by the 1:1 reaction of the corresponding homo-carboxylato complexes **25**, **26**, and [${\text{Mn}}_{8}^{\text{III}}$${\text{Mn}}_{4}^{\text{IV}}$O_12_(O_2_CCHCl_2_)_16_(H_2_O)_4_] (**29**), the latter prepared by the reaction of **1** with a large excess of CHCl_2_CO_2_H in CH_2_Cl_2_ and removal of MeCO_2_H as its toluene azeotrope, Equation (29). This method involves ligand redistribution and the isolated products are the 8:8 complexes. Complexes **27** and **28** are SMMs with *U*_eff_ values of ~50 cm^−1^.

(25) $$\begin{array}{l} \left[{\text{Mn}}_{8}^{\text{III}}{\text{Mn}}_{4}^{\text{IV}}{\text{O}}_{12}{\left({\text{O}}_{2}{\text{CCH}}_{2}{}^{\text{t}}\text{Bu}\right)}_{16}{\left({\text{H}}_{2}\text{O}\right)}_{4}\right]+8\;{\text{CHCl}}_{2}{\text{CO}}_{2}\text{H }\\ \;\overset{{\text{CH}}_{2}{\text{Cl}}_{2}}{\to }[{\text{Mn}}_{8}^{\text{III}}{\text{Mn}}_{4}^{\text{IV}}{\text{O}}_{12}{\left({\text{O}}_{2}{\text{CCHCl}}_{2}\right)}_{8}{\left({\text{O}}_{2}{\text{CCH}}_{2}{}^{\text{t}}\text{Bu}\right)}_{8}\\ \;{\left({\text{H}}_{2}\text{O}\right)}_{3}]+8\;{\text{Bu}}^{\text{t}}\;{\text{CH}}_{2}{\text{CO}}_{2}\text{H}+{\text{H}}_{2}\text{O} \end{array}$$

(26) $$\begin{array}{l} \left[{\text{Mn}}_{8}^{\text{III}}{\text{Mn}}_{4}^{\text{IV}}{\text{O}}_{12}{\left({\text{O}}_{2}\text{CEt}\right)}_{16}{\left({\text{H}}_{2}\text{O}\right)}_{4}\right]+8\;{\text{CHCl}}_{2}{\text{CO}}_{2}\text{H}\\ \;\overset{{\text{CH}}_{\text{2}}{\text{Cl}}_{\text{2}}}{\to }\left[{\text{Mn}}_{\text{8}}^{\text{III}}{\text{Mn}}_{\text{4}}^{\text{IV}}{\text{O}}_{\text{12}}{\left({\text{O}}_{2}{\text{CCHCl}}_{\text{2}}\right)}_{\text{8}}{\left({\text{O}}_{2}\text{CEt}\right)}_{\text{8}}{\left({\text{H}}_{\text{2}}\text{O}\right)}_{\text{3}}\right]\;\\ \;+\text{ 8 }{\text{EtCO}}_{2}\text{H}+{\text{H}}_{\text{2}}\text{O} \end{array}$$

(27) $$\begin{array}{l} \left[{\text{Mn}}_{8}^{\text{III}}{\text{Mn}}_{4}^{\text{IV}}{\text{O}}_{12}{\left({\text{O}}_{2}{\text{CCHCl}}_{2}\right)}_{16}{\left({\text{H}}_{2}\text{O}\right)}_{4}\right]+[{\text{Mn}}_{8}^{\text{III}}{\text{Mn}}_{4}^{\text{IV}}{\text{O}}_{12}\\ \;{\left({\text{O}}_{2}{\text{CCH}}_{2}{}^{\text{t}}\text{Bu}\right)}_{16}{\left({\text{H}}_{2}\text{O}\right)}_{3}]\;\overset{{\text{CH}}_{2}{\text{Cl}}_{2}}{\to }\;2\;[{\text{Mn}}_{8}^{\text{III}}{\text{Mn}}_{4}^{\text{IV}}{\text{O}}_{12}\;\\ \;{\left({\text{O}}_{2}{\text{CCHCl}}_{2}\right)}_{8}\;{\left({\text{O}}_{2}{\text{CCH}}_{2}{}^{\text{t}}\text{Bu}\right)}_{8}{\left({\text{H}}_{2}\text{O}\right)}_{3}]\;+\;{\text{H}}_{2}\text{O} \end{array}$$

(28) $$\begin{array}{l} \left[{\text{Mn}}_{8}^{\text{III}}{\text{Mn}}_{4}^{\text{IV}}{\text{O}}_{12}{\left({\text{O}}_{2}{\text{CCHCl}}_{2}\right)}_{16}{\left({\text{H}}_{2}\text{O}\right)}_{4}\right]+[{\text{Mn}}_{8}^{\text{III}}{\text{Mn}}_{4}^{\text{IV}}{\text{O}}_{12}\\ \;{\left({\text{O}}_{2}\text{CEt}\right)}_{\text{16}}{\left({\text{H}}_{\text{2}}\text{O}\right)}_{\text{3}}]\;\overset{{\text{CH}}_{\text{2}}{\text{Cl}}_{\text{2}}}{\to }\text{ 2 }[{\text{Mn}}_{\text{8}}^{\text{III}}{\text{Mn}}_{\text{4}}^{\text{IV}}{\text{O}}_{\text{12}}{\left({\text{O}}_{\text{2}}{\text{CCHCl}}_{\text{2}}\right)}_{\text{8}}\;\\ \;{\left({\text{O}}_{\text{2}}\text{CEt}\right)}_{\text{8}}{\left({\text{H}}_{\text{2}}\text{O}\right)}_{\text{3}}]\text{ + }{\text{H}}_{\text{2}}\text{O} \end{array}$$

(29) $$\begin{array}{l} \left[{\text{Mn}}_{8}^{\text{III}}{\text{Mn}}_{4}^{\text{IV}}{\text{O}}_{12}{\left({\text{O}}_{2}\text{CMe}\right)}_{16}{\left({\text{H}}_{2}\text{O}\right)}_{4}\right]+16\;{\text{CHCl}}_{2}{\text{CO}}_{2}\text{H }\\ \;\overset{{\text{CH}}_{2}{\text{Cl}}_{2}}{\to }\;\left[{\text{Mn}}_{8}^{\text{III}}{\text{Mn}}_{4}^{\text{IV}}{\text{O}}_{12}{\left({\text{O}}_{2}{\text{CCHCl}}_{2}\right)}_{16}{\left({\text{H}}_{2}\text{O}\right)}_{4}\right]\;+16\;{\text{MeCO}}_{2}\text{H} \end{array}$$

### Substitution of carboxylato ligands by non-carboxylate groups

Work from Christou's group has led to the development of procedures for partial (and sometimes site-selective) non-carboxylato substitution in {${\text{Mn}}_{8}^{\text{III}}$${\text{Mn}}_{4}^{\text{IV}}$} SMMs. Site-selective carboxylato abstraction has been become possible from [${\text{Mn}}_{8}^{\text{III}}$${\text{Mn}}_{4}^{\text{IV}}$O_12_(O_2_CR)_16_(H_2_O)_4_] (R = Ph, **17**; R = Bu^*t*^CH_2_, **25**) by treatment with 4 equivs of HNO_3_ in MeCN, Equation (30). The products are [${\text{Mn}}_{8}^{\text{III}}$${\text{Mn}}_{4}^{\text{IV}}$O_12_(O_2_CR)_12_(NO_3_)_4_(H_2_O)_4_] (R = Ph, **30**; R = Bu^*t*^CH_2_, **31**). The four nitrato groups are coordinated in the same 2.11 mode as the carboxylato ligands at the type **I** (section Substitution of Carboxylato Ligands by Other Carboxylato Groups) Mn^III^ axial Jahn-Teller positions (Artus et al., 2001). The reaction represented by Equation (30) can be reversed; reactions of **30** and **31** with 4 equivs of NaO_2_CR in CH_2_Cl_2_/MeOH give complexes **17** and **25**, respectively, Equation (31). This makes complexes such as **30** and **31** candidates for reactivity centered at the nitrato positions, taking advantage of the good leaving properties of bound ${\text{NO}}_{3}^{-}$ ions. Complex **31** (*S* = 10) is an SMM with a *U*_eff_ value of 50.0 cm^−1^. The complex also shows hysteresis in magnetization vs. dc field scans; the hysteresis loops show steps at regular intervals of magnetic field, the diagnostic evidence of QTM.

(30) $$\begin{array}{l} \left[{\text{Mn}}_{8}^{\text{III}}{\text{Mn}}_{4}^{\text{IV}}{\text{O}}_{12}{\left({\text{O}}_{2}\text{CR}\right)}_{16}{\left({\text{H}}_{2}\text{O}\right)}_{4}\right]+4\;{\text{HNO}}_{3}\\ \;\overset{\text{MeCN}}{\to }\left[{\text{Mn}}_{8}^{\text{III}}{\text{Mn}}_{4}^{\text{IV}}{\text{O}}_{12}{\left({\text{O}}_{2}\text{CR}\right)}_{12}{\left({\text{NO}}_{3}\right)}_{4}{\left({\text{H}}_{2}\text{O}\right)}_{4}\right]+4\;{\text{RCO}}_{2}\text{H} \end{array}$$

(31) $$\begin{array}{l} \left[{\text{Mn}}_{8}^{\text{III}}{\text{Mn}}_{4}^{\text{IV}}{\text{O}}_{12}{\left({\text{O}}_{2}\text{CR}\right)}_{12}{\left({\text{NO}}_{3}\right)}_{4}{\left({\text{H}}_{2}\text{O}\right)}_{4}\right]+4\;{\text{NaO}}_{2}\text{CR}\\ \;\overset{{\text{CH}}_{2}{\text{Cl}}_{2}/\text{MeOH}}{\to }\left[{\text{Mn}}_{8}^{\text{III}}{\text{Mn}}_{4}^{\text{IV}}{\text{O}}_{12}{\left({\text{O}}_{2}\text{CR}\right)}_{16}{\left({\text{H}}_{2}\text{O}\right)}_{4}\right]+4\;{\text{NaNO}}_{3} \end{array}$$

In a similar way, the reaction of **1** with 8 equivs of benzenesulfonic acid (PhSO_3_H) in MeCN, followed by several cycles of the removal of generated MeCO_2_H as the toluene azeotrope to ensure complete reaction, gives complex [${\text{Mn}}_{8}^{\text{III}}$${\text{Mn}}_{4}^{\text{IV}}$O_12_(O_2_CMe)_8_(O_3_SPh)_8_(H_2_O)_4_] (**32**) in a >95% yield, Equation (32) (Chakov et al., 2003). The Ph${\text{SO}}_{3}^{-}$ ligands are at the eight axial (**I** and **I′**, see in the “Substitution of Carboxylato Ligands by Other Carboxylato Groups” section above) Mn^III^ Jahn-Teller positions, again as expected on the basis of relative basicities (p*K*_a_ of MeCO_2_H = 4.76, p*K*_a_ of PhSO_3_H = 2.55). In other words, the more basic (stronger donors) ${\text{MeCO}}_{2}^{-}$ ligands prefer the equatorial sites where shorter, stronger Mn-O bonds can be formed stabilizing better the molecule. The less basic Ph${\text{SO}}_{3}^{-}$ ligands are located at axial positions where they bridge either Mn^III^/Mn^III^ or Mn^III^/Mn^IV^ pairs and therefore have one or both of their O atoms on the Jahn-Teller elongation axes of the Mn^III^ atoms. The mixed-ligand cluster **32** retains both the high ground-state spin (*S* = 10) and SMM behavior (*U*_eff_ = ~47 cm^−1^) of the parent SMM **1** and its carboxylato derivatives. Hysteresis loops were observed below 4.0 K; their coercivities increase with decreasing temperature, a typical behavior of SMMs. The synthetic importance of reactions, such as that represented by Equation (32), is that regioselective chemistry at the axial positions with anionic ligands can become a reality.

(32) $$\begin{array}{l} \left[{\text{Mn}}_{8}^{\text{III}}{\text{Mn}}_{4}^{\text{IV}}{\text{O}}_{12}{\left({\text{O}}_{2}\text{CMe}\right)}_{16}{\left({\text{H}}_{2}\text{O}\right)}_{4}\right]+8\;{\text{PhSO}}_{3}\text{H }\\ \;\overset{\text{MeCN}}{\to }\;\left[{\text{Mn}}_{8}^{\text{III}}{\text{Mn}}_{4}^{\text{IV}}{\text{O}}_{12}{\left({\text{O}}_{2}\text{CMe}\right)}_{8}{\left({\text{O}}_{3}\text{SPh}\right)}_{8}{\left({\text{H}}_{2}\text{O}\right)}_{4}\right]\\ \;+8\;{\text{MeCO}}_{2}\text{H} \end{array}$$

In the non-carboxylato substitution of {${\text{Mn}}_{8}^{\text{III}}$${\text{Mn}}_{4}^{\text{IV}}$} SMMs i.e., **1** and its derivatives, sometimes steric effects overcome basicity effects. A typical example is the reaction of **1** with 8 equivs of diphenylphosphinic acid (Ph_2_PO_2_H) in MeCN that gives the 8:8 cluster [${\text{Mn}}_{8}^{\text{III}}$${\text{Mn}}_{4}^{\text{IV}}$O_12_(O_2_CMe)_8_(O_2_PPh_2_)_8_(H_2_O)_4_] (**33**) in 60% yield. In **33**, the 4 ${\text{MeCO}}_{2}^{-}$ ligands are located at the four axial Mn^III^/Mn^III^ and 4 of the eight equatorial Mn^III^/Mn^III^ carboxylate sites have been replaced by Ph_2_${\text{PO}}_{2}^{-}$ groups, while the remaining equatorial sites and the four axial Mn^III^/Mn^IV^ sites remain occupied by ${\text{MeCO}}_{2}^{-}$ ligands (Boskovic et al., 2001). In other words, the large steric bulk of the Ph_2_${\text{PO}}_{2}^{-}$ ligands has, as a consequence, their equal distribution between axial and equatorial sites. The SMM properties are retained in **33** (*U*_eff_ = ~42 cm^−1^). Magnetization hysteresis loops were observed for oriented crystals of **33**·12CH_2_Cl_2_.

### Reduction pathways for the {${\text{Mn}}_{8}^{\text{III}}$${\text{Mn}}_{4}^{\text{IV}}$} SMMs

Detailed electrochemical studies on many members of the {${\text{Mn}}_{8}^{\text{III}}$${\text{Mn}}_{4}^{\text{IV}}$} family of SMMs (**1**, **17**, **25**, **26**,…) have revealed several oxidation and reduction processes, with some of the latter being reversible. The oxidation processes are at rather high potentials, but the reduction processes are accessible by chemical means. Electron-withdrawing R groups in the carboxylato ligands (e.g., R = CHCl_2_ or C_6_F_5_) favor the reduction processes, because they reduce the electron density in the core and make reductions easier. An ideal reducing agent is the iodide (I^−^) because: (i) Its reducing strength is sufficient, but mild; (ii) there are many organic salts C^+^I^−^, the majority of which have good solubility in a variety of organic solvents; and (iii) the only by-product of the redox processes is elemental I_2_ which can be easily removed.

**Figure 8:**
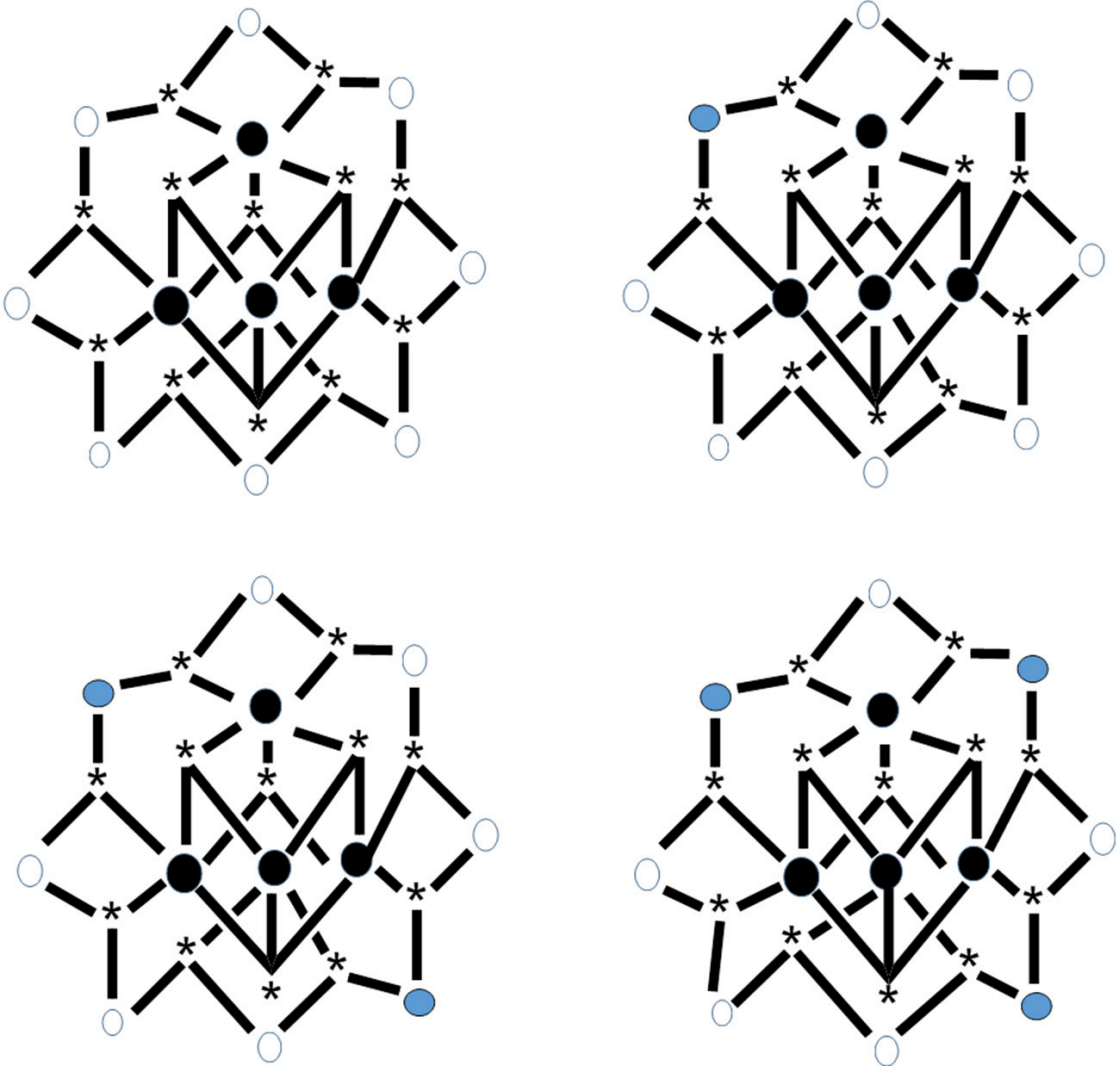
The crystallographically established {Mn_12_O_12_} cores of the {Mn_12_}, {Mn_12_}^−^, and {Mn_12_}^2−^ SMMs, and proposed core for the {Mn_12_}^3−^ SMMs. The symbol 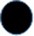 represents Mn^IV^, the symbol 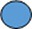 represents Mn^III^, the symbol 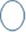 represents Mn^II^, and the asterisk illustrates the triply bridging O^2−^ group (Schematic drawing inspired by Bagai and Christou, 2009). Coordination bonds are drawn in bold.

Reduction of complex **26** with 1 equiv of Ph_4_PI in CH_2_Cl_2_ leads to complex (Ph_4_P)[Mn^II^${\text{Mn}}_{7}^{\text{III}}$${\text{Mn}}_{4}^{\text{IV}}$O_12_(O_2_CEt)_16_(H_2_O)_4_] (**34**) in ~70% yield, Equation (33). The structure reveals that the extra electron is localized on an outer (originally Mn^III^) ion rather than an inner (cubane) Mn^IV^ center (see Figure 8), and the product is a trapped-valence {Mn^II^${\text{Mn}}_{7}^{\text{III}}$${\text{Mn}}_{4}^{\text{IV}}$}^−^ ion (Eppley et al., 1995). Cluster **34** has an *S* = 19/2 ground state being SMM. The complex exhibits well-developed hysteresis loops. The benzoate analog of **34**, i.e., cluster (Ph_4_P)[Mn_12_O_12_(O_2_CPh)_16_(H_2_O)_4_] (**35**) can be prepared in a similar way (Aubin et al., 1999). For the eight “external” Mn ions (formerly Mn^III^), it was not possible to decide whether a trapped-valence {Mn^II^${\text{Mn}}_{7}^{\text{III}}$} or an electronically delocalized description is the most scientifically correct. Complexes such as **34** and **35** (both with *S* = 19/2) are ideal candidates for the study of QTM in half-integer spin systems; QTM is not allowed in such compounds in the absence of an applied magnetic field.

(33) $$\begin{array}{l} {}[{\text{Mn}}_{8}^{\text{III}}{\text{Mn}}_{4}^{\text{IV}}{\text{O}}_{12}{\left({\text{O}}_{2}\text{CEt}\right)}_{16}{\left({\text{H}}_{2}\text{O}\right)}_{3}]+{\text{Ph}}_{4}\text{PI}+{\text{H}}_{2}\text{O }\\ \;\overset{{\text{CH}}_{2}{\text{Cl}}_{2}}{\to }({\text{Ph}}_{4}\text{P})[{\text{Mn}}^{\text{II}}{\text{Mn}}_{7}^{\text{III}}{\text{Mn}}_{4}^{\text{IV}}{\text{O}}_{12}{\left({\text{O}}_{2}\text{CEt}\right)}_{16}{\left({\text{H}}_{2}\text{O}\right)}_{4}]\;\\ \;+1/2\;{\text{I}}_{2} \end{array}$$

The carboxylato substitution reactions described in section Substitution of Carboxylato Ligands by Other Carboxylato Groups have permitted the incorporation onto the {Mn_12_} unit of carboxylate ligands possessing strongly electron-withdrawing groups, and these can raise the reduction potentials so that the second reduction is feasible with I^−^. For example, the reaction of **29** with two equivs of Ph_4_PI in MeCN provides access to complex (Ph_4_P)_2_[${\text{Mn}}_{2}^{\text{II}}$${\text{Mn}}_{6}^{\text{III}}$${\text{Mn}}_{4}^{\text{IV}}$O_12_(O_2_CCHCl_2_)_16_(H_2_O)_4_] (**36**) in very good yield (Soler et al., 2003), Equation (34). Crystallization of the bulk material from CH_2_Cl_2_/hexanes yields a mixture of two crystals forms, **36**·4CH_2_Cl_2_·H_2_O (**36a**) and **36**·6CH_2_Cl_2_ (**36b**), both of which have been structurally characterized by single-crystal, X-ray crystallography. The molecular structures are almost identical, with the two extra electrons localized on former Mn^III^ centers (see Figure 8) generating a trapped-valence {${\text{Mn}}_{2}^{\text{II}}$${\text{Mn}}_{6}^{\text{III}}$${\text{Mn}}_{4}^{\text{IV}}$}^2−^ ion. Bulk dc magnetization data for dried **36** in the 1.8–4.0 K and 1–7 T ranges were fit to give *S* = 10, *D* = −0.275 cm^−1^ and *g* = 2.00. The *U*_eff_ values for **36a** and **36b** are ~13 and ~21 cm^−1^, respectively. Magnetization vs. dc field scans on single crystals of the two samples gave hysteresis loops containing steps due to QTM. The step separations yielded |*D*|/*g* values of 0.087 and 0.14 cm^−1^ for **36a** and **36b**, respectively, indicating that the differences in *U*_eff_ are caused by changes to *D*.

(34) $$\begin{array}{l} {}[{\text{Mn}}_{8}^{\text{III}}{\text{Mn}}_{4}^{\text{IV}}{\text{O}}_{12}{\left({\text{O}}_{2}{\text{CCHCl}}_{2}\right)}_{16}{\left({\text{H}}_{2}\text{O}\right)}_{4}]+2\;{\text{Ph}}_{4}\text{PI}\overset{\text{MeCN}}{\to }\\ \;{\left({\text{Ph}}_{4}\text{P}\right)}_{2}[{\text{Mn}}_{2}^{\text{II}}{\text{Mn}}_{6}^{\text{III}}{\text{Mn}}_{4}^{\text{IV}}{\text{O}}_{12}{\left({\text{O}}_{2}{\text{CCHCl}}_{2}\right)}_{16}{\left({\text{H}}_{2}\text{O}\right)}_{4}]+{\text{I}}_{2} \end{array}$$

The electrochemical properties of **29** exhibit a third reversible reduction accessible to I^−^. Thus, the {Mn_12_} family of SMMs has been extended to a fourth isolated member. The reaction of **29** with 3 equivs of Pr^*n*^_4_NI or Me_4_NI in MeCN for a long time (~2 days) give complexes (Pr^*n*^_4_N)_3_[${\text{Mn}}_{3}^{\text{II}}$${\text{Mn}}_{5}^{\text{III}}$${\text{Mn}}_{4}^{\text{IV}}$O_12_(O_2_CCHCl_2_)_16_(H_2_O)_4_] (**37**) and (Me_4_N)_3_[${\text{Mn}}_{3}^{\text{II}}$${\text{Mn}}_{5}^{\text{III}}$${\text{Mn}}_{4}^{\text{IV}}$O_12_(O_2_CCHCl_2_)_16_(H_2_O)_4_] (**38**), respectively (Bagai and Christou, 2007), Equation (35) (C = Pr^*n*^_4_N, Me_4_N); the yields are high (~80%). The complexes are not stable in solution and all the attempts to grow single-crystals of the salts were unsuccessful. Dc magnetization data on dried microcrystalline samples were fit by matrix diagonalization methods to give *S* = 17/2, *D* = −0.25 cm^−1^ and *g* = 1.91 for **37** and *S* = 17/2, *D* = −0.23 cm^−1^ and *g* = 1.90 for **38**. The two complexes exhibit frequency-dependent out-of-phase magnetic susceptibility signals at ≤2.5 K, indicating them to be SMMs, albeit at lower temperatures compared with the {Mn_12_} SMMs (6–8 K range), {Mn_12_}^−^ SMMs (4–6 K range), and {Mn_12_}^2−^ SMMs (2–4 K range); the shifts to lower temperatures are indicative of the decreasing *S* and *D*-values upon successive reduction and therefore the decreasing *U*_eff_ value. The decreased |*D*| values in the order {Mn_12_} >{Mn_12_}^−^ >{Mn_12_}^2−^ >{Mn_12_}^3−^ indicate that the third added electron is localized on a formerly Mn^III^ ion, since the Jahn-Teller-distorted Mn^III^ ions are the main source of the molecular anisotropy (see Figure 8).

(35) $$\begin{array}{l} {}[{\text{Mn}}_{8}^{\text{III}}{\text{Mn}}_{4}^{\text{IV}}{\text{O}}_{12}{\left({\text{O}}_{2}{\text{CCHCl}}_{2}\right)}_{16}{\left({\text{H}}_{2}\text{O}\right)}_{4}]+3\;{\text{C}}^{+}{\text{I}}^{-}\;\overset{\text{MeCN}}{\to }\\ \;{\text{C}}_{3}[{\text{Mn}}_{3}^{\text{II}}{\text{Mn}}_{5}^{\text{III}}{\text{Mn}}_{4}^{\text{IV}}{\text{O}}_{12}{\left({\text{O}}_{2}{\text{CCHCl}}_{2}\right)}_{16}{\left({\text{H}}_{2}\text{O}\right)}_{4}]+3\;{\text{I}}_{2} \end{array}$$

The preferential reduction of Mn^III^ rather than Mn^IV^ can be interpreted in terms of the different environments which these metals have within the {Mn_12_} unit. Thus: (i) The Mn^IV^ centers are each bound to five hard O^2−^ groups which favor higher oxidation states and preclude reduction to Mn^III^; and (ii) the reduction of a Mn^IV^ would generate a Mn^III^ center whose Jahn-Teller distortion axis would have to include at least one Mn^III^-O^2−^ bond (an inherently strong bond) and would introduce a severe strain into the rigid {${\text{Mn}}_{4}^{\text{IV}}$O_4_} subcore.

### “Switching on” SMM behavior through substitution of bridging hydroxido groups by end-on azido or isocyanato ligands in pre-formed clusters

One of the prerequisities of a 3d-metal cluster to behave as SMM is a high or a relatively high total spin in the ground state. The high-spin ground state can result from either ferromagnetic (or ferrimagnetic) metal···metal exchange interactions and/or topologically frustrated antiferromagnetic exchange interactions. In general, however, it is difficult to predict which topology will lead to high-spin ground states, and more difficult to design and achieve the syntheses of such compounds. In many coordination clusters, the exchange interactions between the paramagnetic metal ions is propagated by hydroxido (OH^−^), oxido (O^2−^), alkoxido or alkoxido-type (RO^−^), or carboxylato (${\text{RCO}}_{2}^{-}$) ligands, or a combination of two or more such ligands. These ligands most often propagate antiferromagnetic exchange interactions. Our group (Papaefstathiou et al., 2001a,b; Boudalis et al., 2004, 2008), in collaboration with Escuer's group, have developed a general strategy for the synthesis of high-spin clusters, which often “switches on” SMM behavior. The strategy is based on the substitution of bridging hydroxido or/and alkoxido groups in pre-formed coordination clusters (generally low-spin due to antiferromagnetic interactions) by EO azido groups (2.20, 3.30, 4.40) or N-bonded cyanato (isocyanato) bridging groups (2.02, 3.03, 4.04). The nuclearity does not change, but the core does. The entering bridging groups introduce ferromagnetic components in the superexchange scheme of the cluster, always increasing the ground-state spin and sometimes “switching on” SMM properties. The method works better for divalent 3d metals. The strategy is illustrated with one example (Boudalis et al., 2004, 2008).

Cluster [${\text{Fe}}_{9}^{\text{II}}$(OH)_2_(O_2_CMe)_8_{(py)_2_CO_2_}_4_] (**39**), where (py)_2_CO is di-2-pyridyl ketone (**C** in Figure 1) and (py)_2_${\text{CO}}_{2}^{2-}$ is the dianion of the *gem*-diol form of (py)_2_CO (**D** in Figure 1) is prepared under an inert atmosphere by the reaction outlined in eqn. (36) in ~30% yield. The nine Fe^II^ atoms adopt the topology of two square pyramids sharing a common apex and are held together by four 5.3311 (py)_2_${\text{CO}}_{2}^{2-}$ ligands (**PP** in Figure 2). Each Fe^II^···Fe^II^ edge of the bases of the pyramids is further bridged by one syn, syn-2.11 ${\text{MeCO}}_{2}^{-}$ group. The four ${\text{MeCO}}_{2}^{-}$ groups create a concave cavity in the base of each pyramid, into which a rare 4.4 OH^−^ group is trapped capping the square base. The magnetic study of **39** reveals an overall antiferromagnetic behavior with an *S* = 2 ground state.

(36) $$\begin{array}{l} 9\;{\text{Fe}}^{\text{II}}{\left({\text{O}}_{2}\text{CMe}\right)}_{2}+4\;{\left(\text{py}\right)}_{2}\text{CO}+6\;{\text{H}}_{2}\text{O}\overset{\text{MeCN},{\text{N}}_{2}}{\underset{\text{T}}{\to }}[{\text{Fe}}_{9}^{\text{II}}{\left(\text{OH}\right)}_{2}\\ \;{\left({\text{O}}_{2}\text{CMe}\right)}_{8}{\left\{{\left(\text{py}\right)}_{2}{\text{CO}}_{2}\right\}}_{4}]+10\;{\text{MeCO}}_{2}\text{H} \end{array}$$

**Figure 9:**
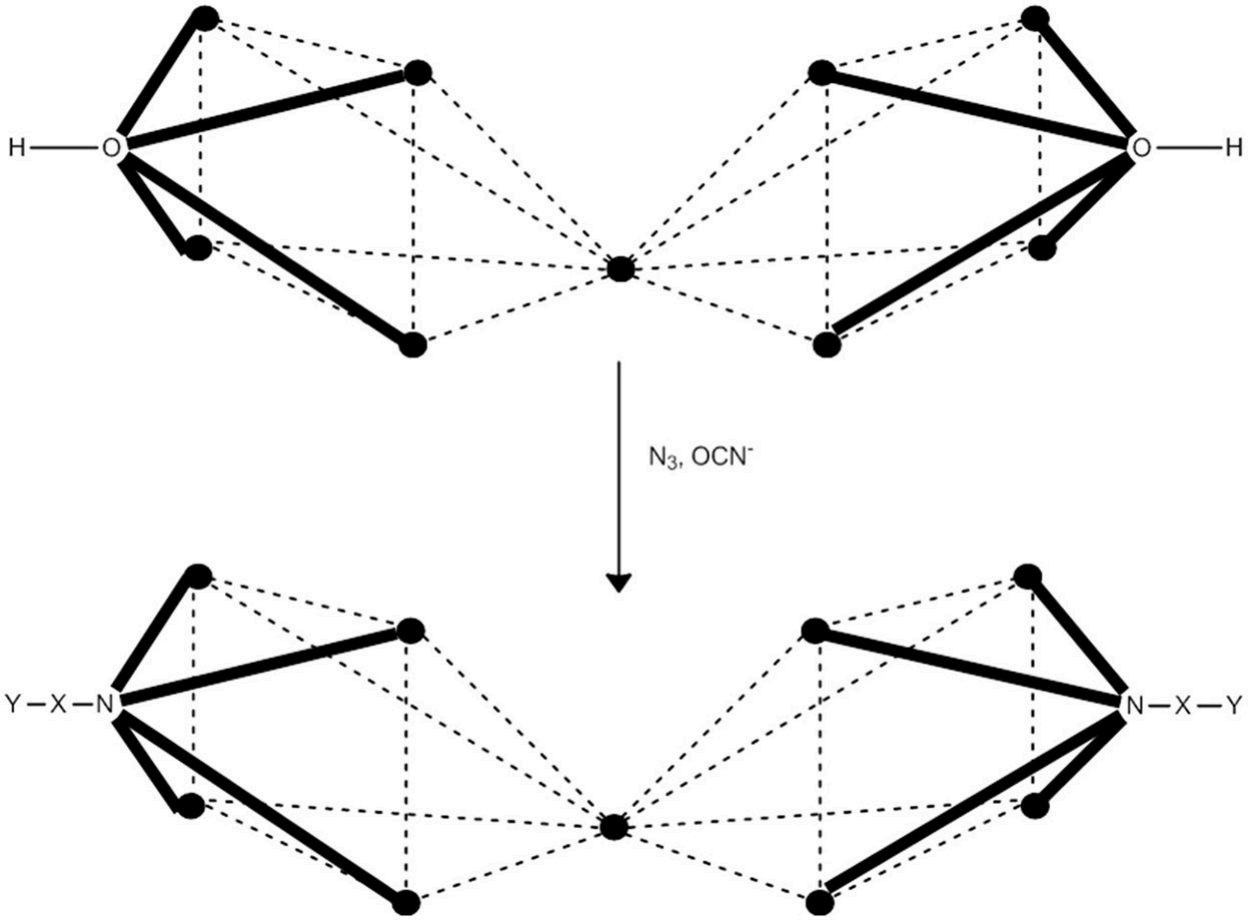
Inorganic core change upon conversion of cluster **39** to the azido- and isocyanato-bridged clusters **40** and **41**. The symbol 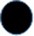 represents Fe^II^. The dashed lines do not represent chemical bonds, but they help the understanding of the metal topology. Coordination bonds are drawn in bold. X, Y = N for **40**; X = C and Y = 0 for **41**.

Anaerobic reactions of **39** with a slight excess of NaN_3_ or KOCN in refluxing MeCN provides access to clusters [${\text{Fe}}_{9}^{\text{II}}$(N_3_)_2_(O_2_CMe)_8_{(py)_2_CO_2_}_4_] (**40**) and [${\text{Fe}}_{9}^{\text{II}}$(NCO)_2_(O_2_CMe)_8_{(py)_2_CO_2_}_4_] (**41**), respectively, in moderate yields, Equation (37). Complexes **40** and **41** have striking structurally similarity to **39**, the main difference being the replacement of the 4.4 OH^−^ groups in the latter by the 4.40 ${\text{N}}_{3}^{-}$ and 4.04 OCN^−^ groups in the products (see Figure 9).^1^H paramagnetic NMR spectroscopic studies in CD_3_CN reveal that the three enneanuclear Fe(II) complexes are stable in solution. Dc magnetic susceptibility studies show that the ground-state spin values are higher in **40** and **41** than in **39**; variable-field experiments have shown that the ground state is not well isolated from the low-lying excited states, and it cannot thus be determined with accuracy. X-band EPR spectroscopy at 4.2 K reveals characteristic integer-spin signals for **39**, but not for the azido- and isocyanato-bridged clusters. Complexes **40** and **41** are SMMs with *U*_eff_ values of 28.5 and 30.5 cm^−1^, respectively. The slow magnetic relaxation has also been observed by ^57^Fe Mössbauer spectroscopy.

(37) $$\begin{array}{l} {}[{\text{Fe}}_{9}^{\text{II}}{\left(\text{OH}\right)}_{2}{\left({\text{O}}_{2}\text{CMe}\right)}_{8}{\left\{{\left(\text{py}\right)}_{2}{\text{CO}}_{2}\right\}}_{4}]+2\;\left({\text{NaN}}_{3},\text{KOCN}\right)\;\\ \;\overset{\text{MeCN},{\text{N}}_{2}}{\underset{\text{T}}{\to }}[{\text{Fe}}_{9}^{\text{II}}{\left({\text{N}}_{3},\text{NCO}\right)}_{2}{\left({\text{O}}_{2}\text{CMe}\right)}_{8}\;{\left\{{\left(\text{py}\right)}_{2}{\text{CO}}_{2}\right\}}_{4}]\;\\ \;+2\left(\text{Na},\text{K}\right)\text{ OH} \end{array}$$

### “Tweaking” the spin

The strategy reported in section “Switching on” SMM Behavior Through Substitution of Bridging Hydroxido Groups by End-on Azido or Isocyanato Ligands in Pre-formed Clusters involves conversion of a low-spin 3d-metal cluster into a high-spin one with the simultaneous “switching on” of SMM properties. This approach changes the core. An alternative approach is to start with a pre-formed, high-spin molecule (and perhaps an SMM) and then perturb it without significant core change by targeted modification of the peripheral ligands, in order to modify some exchange parameters, and slightly increase the ground-state spin and “switch on” or enhance the SMM properties. This strategy has been termed “spin tweaking” (Stamatatos et al., 2007a,b). We give an example of this approach from Mn chemistry.

**Figure 10:**
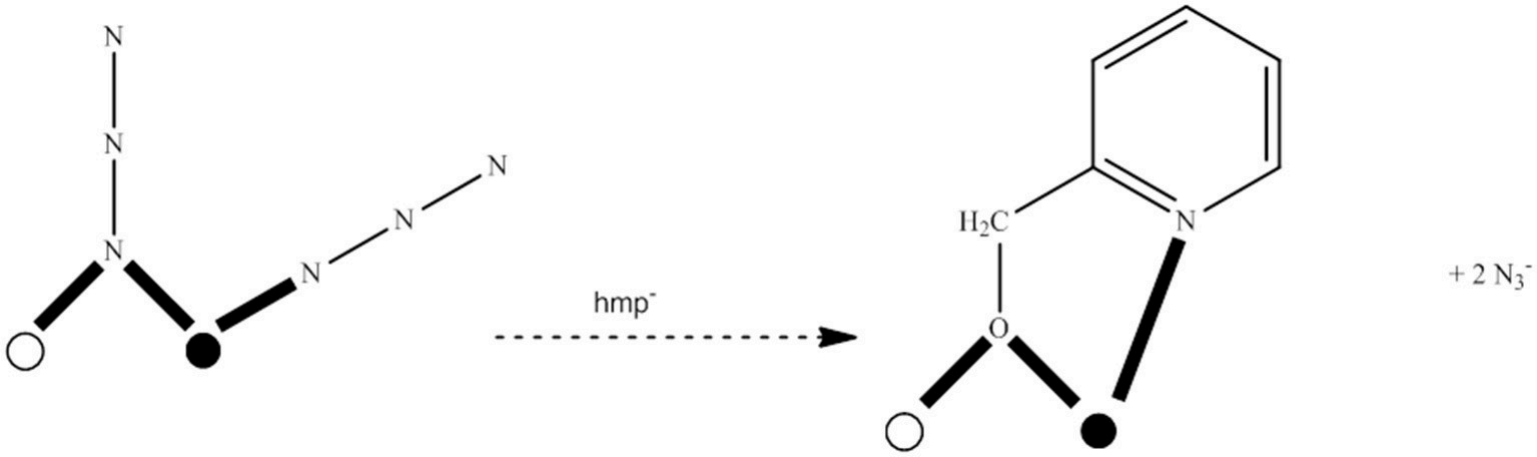
Schematic drawing of the synthetic approach that leads to the **42**→**43** conversion. The symbols 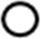 and 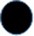 represent Mn^II^ and Mn^III^, respectively. Coordination bonds are drawn in bold.

The starting material is [${\text{Mn}}_{6}^{\text{II}}$${\text{Mn}}_{18}^{\text{III}}$Mn^IV^(OH)_2_O_18_(N_3_)_12_(pdm)_6_(pdmH)_6_](N_3_)_2_ (**42**) (Murugesu et al., 2004, 2008), where pdm^2−^ and pdmH^−^ are the di- and the monoanion, respectively, of pyridine-2,6-dimethanol (pdmH_2_; **R** in Figure 1). The core is held together by 12 4.4 O^2−^, 6 3.3 O^2−^ and two 3.3 OH^−^ groups, as well as 6 2.20 ${\text{N}}_{3}^{-}$ ions and the deprotonated alkoxido arms of the 5.331 pdm^2−^ (**QQ** in Figure 2) and 3.211 pdmH^−^ ligands (**RR** in Figure 2). The complex has a total spin of *S* = 51/2 in the ground state, being an SMM (*U*_eff_ = ~8 cm^−1^). The target was to replace the peripheral 6 2.20 and 6 terminal (1.10) azido ligands by 6 2.21 hmp^−^ ligands (**SS** in Figure 2), where hmp^−^ is the monoanion of 2-(hydroxymethyl)pyridine (hmpH; **S** in Figure 1). The synthetic idea was that the bridging deprotonated oxygen atom of hmp^−^ (known to promote ferromagnetic exchange between Mn ions) can be considered as the equivalent of the bridging N atom of the 2.20 ${\text{N}}_{3}^{-}$ ligands, while the 2-pyridyl nitrogen atom of hmp^−^ is the equivalent of the terminal N atom of the 1.10 ${\text{N}}_{3}^{-}$ ligands. The reaction of **42**, Na(hmp) and NaClO_4_ in 1:6:6 molar ratio in MeCN/MeOH gives a dark brown solution, from which is subsequently crystallized [${\text{Mn}}_{6}^{\text{II}}$${\text{Mn}}_{18}^{\text{III}}$Mn^IV^(OH)(OMe)O_18_(hmp)_6_(pdm)_6_(pdmH)_6_](N_3_)_2_(ClO_4_)_6_ (**43**) in ~65% yield, Equation (38). The crystal structure shows the core of **43** to be isostructural with that of **42**. The main differences are that the 12 coordinated azido groups of **42** are replaced by 6 2.21 hmp^−^ ligands in **43** (see Figure 10) and also a 2.2 OH^−^ group of the former is substituted by a 2.2 MeO^−^ group in the latter. The result is that all bridging atoms in the product are oxygen atoms and there are thus changes in many exchange interactions. The ground-state spin increases from *S* = 51/2 in **42** to *S* = 61/2 in **43**. Despite the intended increase of *S*, **43** is not a better SMM compared to **42** (Stamatatos et al., 2007a,b). The conversion of **42** to **43** is the first time in which removal of EO azido groups leads to an increase in the ground-state *S*-value in a 3d-metal coordination cluster; usually their addition is a method of increasing the *S* value (section “Switching on” SMM Behavior through Substitution of Bridging Hydroxido Groups by End-on Azido or Isocyanato Ligands in Pre-formed Clusters).

(38) $$\begin{array}{l} {}[{\text{Mn}}_{6}^{\text{II}}{\text{Mn}}_{18}^{\text{III}}{\text{Mn}}^{\text{IV}}{\left(\text{OH}\right)}_{2}{\text{O}}_{18}{\left({\text{N}}_{3}\right)}_{12}{\left(\text{pdm}\right)}_{6}{\left(\text{pdmH}\right)}_{6}]{\left({\text{N}}_{3}\right)}_{2}\;\\ \;+6\text{ Na}\left(\text{hmp}\right)+6\;{\text{NaClO}}_{4}+\text{MeOH}\overset{\text{MeCN}/\text{MeOH}}{\to }\\ \left[{\text{Mn}}_{6}^{\text{II}}{\text{Mn}}_{18}^{\text{III}}{\text{Mn}}^{\text{IV}}\left(\text{OH}\right)\left(\text{OMe}\right){\text{O}}_{18}{\left(\text{hmp}\right)}_{6}{\left(\text{pdm}\right)}_{6}{\left(\text{pdmH}\right)}_{6}\right]\\ {\left({\text{N}}_{3}\right)}_{2}{\left({\text{ClO}}_{4}\right)}_{6}+12\;{\text{NaN}}_{3}+{\text{H}}_{2}\text{O} \end{array}$$

### “Switching on” SMM properties upon conversion of low-spin complexes into high-spin ones without changing the core

The strategy described in section “Switching on” SMM Behavior through Substitution of Bridging Hydroxido Groups by End-on Azido or Isocyanato Ligands in Pre-formed Clusters involves structural change of the core, while in the approach detailed in section “Tweaking” the Spin the core remains the same but the starting materials are already high-spin complexes. A distinctly different approach within the general strategy of modifying known non-SMM complexes is the conversion of certain low-spin complexes into high-spin molecules without change of the core; this conversion results often in the appearance of SMM properties. Our group, in collaboration with Christou's group have contributed into this approach. As mentioned in section Simple 3d-Metal Carboxylates and Carboxylate Triangles and Butterflies as Starting Materials, triangular oxido-centered complexes of the general formulation [Mn_3_O(O_2_CR)_6_L_3_]^0, +^ are useful starting materials in Mn carboxylate chemistry. Antiferromagnetic exchange interactions within the core lead to small *S* values, and the complexes are therefore non-SMMs. It was believed that this common triangular topology could never lead to SMMs. However, it has now been well established that relatively small, ligand-induced structural distortions of the core can alter the sign of the exchange interactions and “switch on” SMM properties.

**Figure 11:**
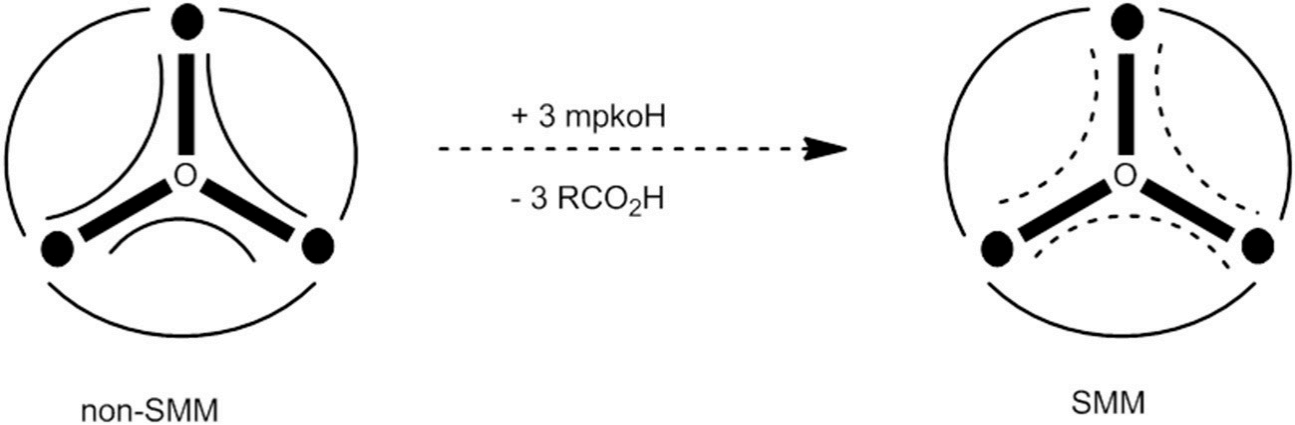
Schematic drawing of the synthetic strategy that leads to the **44**→**45** conversion and the ”switching on” of SMM properties. The symbol 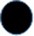 represents Mn^III^. The curved solid and dashed lines represent triatomic carboxylate and diatomic oximato bridging groups, respectively. Coordination Mn^III^-O^2−^ bonds are drawn in bold.

The reactions between the oxido-centered, triangular complexes [${\text{Mn}}_{3}^{\text{III}}$O(O_2_CR)_6_(py)_3_](ClO_4_) [R = Me (**44a**), Et (**44b**), and Ph (**44c**)] and 3 equivs of methyl 2-pyridyl ketoxime (mpkoH; **H** with R = Me in Figure 1) in MeCN/MeOH give complexes [${\text{Mn}}_{3}^{\text{III}}$O(O_2_CR)_3_(mpko)_3_](ClO_4_) [R = Me (**45a**), Et (**45b**), and Ph (**45c**)] in almost quantitative yields (Stamatatos et al., 2005, 2007c), Equation (39). The 1:3 ratio was chosen to allow for the addition of one mpko^−^ ligand onto each edge of the triangle. The process is a simple substitution reaction involving replacement of 3 ${\text{RCO}}_{2}^{-}$ groups and 3 py ligands by three mpko^−^ ones. As in **44a**–**44c**, the cations of **45a**–**45c** contain a {${\text{Mn}}_{3}^{\text{III}}$(μ_3_-O)}^7+^ triangular core, but with each ${\text{Mn}}_{2}^{\text{III}}$ edge now bridged by a 2.11 ${\text{RCO}}_{2}^{-}$ group and a diatomic oximato group of one mpko^−^ ligand (see Figure 11). The 3 ${\text{RCO}}_{2}^{-}$ groups lie on one side of the {${\text{Mn}}_{3}^{\text{III}}$} plane and the three oximato groups on the other. The 2.111 coordination mode of the mpko^−^ ligands (**MM** with R = Me and M = Mn^III^ in Figure 2) results in a buckling of the formerly planar {${\text{Mn}}_{3}^{\text{III}}$(μ_3_-O)}^7+^ core; the consequences are a relative twisting of the three metal octahedra and the displacement of the central O^2−^ group ~0.3 Å above the {${\text{Mn}}_{3}^{\text{III}}$} plane on the same side as the ${\text{RCO}}_{2}^{-}$ groups. The Mn^III^···Mn^III^ exchange interactions are ferromagnetic in **45a**–**45c** resulting in an *S* = 6 ground state. The complexes are SMMs with *U*_eff_ values of ~8 cm^−1^. Magnetization vs. dc field experiments on single crystals of **45a**·3CH_2_Cl_2_ display hysteresis loops that exhibit steps due to QTM. The loops are temperature-independent below 0.3 K, suggesting only QTM in the ground state between the lowest-lying *M*_*S*_ = ±6 levels. High-frequency EPR spectra of single crystals of **45a**·3CH_2_Cl_2_ provide strong evidence for a non-negligible transverse anisotropy of *E* ≥ 0.015 cm^−1^.

(39) $$\begin{array}{l} {}[{\text{Mn}}_{3}^{\text{III}}\text{O}{\left({\text{O}}_{2}\text{CR}\right)}_{6}{\left(\text{py}\right)}_{3}]\left({\text{ClO}}_{4}\right)+3\text{ mpkoH}\overset{\text{MeCN}/\text{MeOH}}{\to }\\ \;[{\text{Mn}}_{3}^{\text{III}}\text{O}{\left({\text{O}}_{2}\text{CR}\right)}_{3}{\left(\text{mpko}\right)}_{3}]\left({\text{ClO}}_{4}\right)+3\;{\text{RCO}}_{2}\text{H}+3\text{ py} \end{array}$$

DFT calculations reveal that the unusual ferromagnetic exchange interactions in complexes **45** may originate from a combination of the “non-planarity” (with respect to the {${\text{Mn}}_{3}^{\text{III}}$} plane) of the bridging oximato groups and the non-parallel alignment of the Jahn-Teller axes; however, several factors are at play (Cano et al., 2008; Atanasov et al., 2011) including the {${\text{Mn}}_{3}^{\text{III}}$} out-of-phase shift of the triply bridging oxido group.

### Ground-state spin switching and enhancing SMM properties via targeted structural distortion

In 2004, our group with the collaboration of Escuer's group reported cluster [${\text{Mn}}_{6}^{\text{III}}$O_2_(O_2_CMe)_2_(sao)_6_(EtOH)_4_] (**46**), where sao^2−^ is the dianion of salicylaldoxime (saoH_2_; **L** with R = H in Figure 1). The complex can be synthesized by the 1:1 reaction between Mn(O_2_CMe)_2_·4H_2_O and saoH_2_ in EtOH at room temperature in moderate yields (~50%), Equation (40). Another route to good yields of pure **46** involves a comproportionation reaction between (Bu^*n*^_4_N)(MnO_4_) and Mn(O_2_CMe)_2_·4H_2_O in the presence of saoH_2_ in EtOH (Milios et al., 2004).

(40) $$\begin{array}{l} 6\;{\text{Mn}}^{\text{II}}{\left({\text{O}}_{\text{2}}\text{CMe}\right)}_{2}\cdot 4\;{\text{H}}_{2}\text{O}+6\;{\text{saoH}}_{2}+4\text{ EtOH}+3/2\;{\text{O}}_{2}\overset{\text{EtOH}}{\to }\;\\ \;[{\text{Mn}}_{6}^{\text{III}}{\text{O}}_{2}{\left({\text{O}}_{2}\text{CMe}\right)}_{2}{\left(\text{sao}\right)}_{6}{\left(\text{EtOH}\right)}_{4}]+10\;{\text{MeCO}}_{2}\text{H }\\ \;+25\;{\text{H}}_{2}\text{O} \end{array}$$

**Figure 12:**
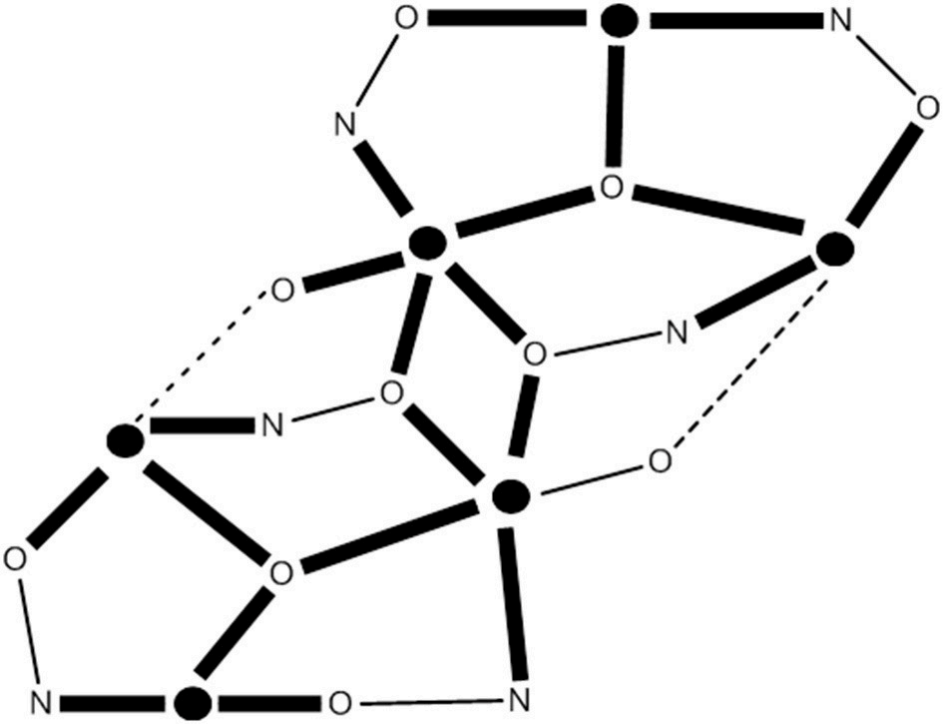
The magnetic core common to the {${\text{Mn}}_{6}^{\text{III}}$} family of SMMs. The symbol 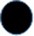 represents Mn^III^. The dashed lines represents the phenolato-o to square-based pyramidal Mn^III^ interaction (Schematic drawing inspired by Milios et al., 2008).

The molecular structure of the complex, which is common to all members of this family (vide infra) consists of two off-set, stacked triangular {${\text{Mn}}_{3}^{\text{III}}$(μ_3_-O)} subunits held together via six sao^2−^ ligands, which span each Mn^III^···Mn^III^ edge of the two triangles, forming Mn^III^-N-O-Mn^III^ bridges, as well as dimerizing the two {${\text{Mn}}_{3}^{\text{III}}$(μ_3_-O)} subunits via their phenolato and oximato O atoms (see Figure 12). The two ${\text{MeCO}}_{2}^{-}$ groups are 2.11 (this also occurs in the firstly reported members of the family with formato, benzoate etc. groups) and sit “above” the plane of the symmetry equivalent triangles. Four of the sao^2−^ ligands show the common 2.111 coordination mode (**LL** with R = H and M = Mn^III^ in Figure 2), while the other two adopt the rather rare 3.211 mode (this is similar to the coordination mode **NN** observed in cluster **24**, section 3.8, with a Mn^III^ atom replacing Na^I^). The core is {${\text{Mn}}_{6}^{\text{III}}$(3.3-O)_2_(3.21-ONR″)_2_(2.11-ONR″)_4_}^8+^, where R″NO^−^ = sao^2−^. The four “central” Mn^III^ ions are 6-coordinate with distorted octahedral geometries, while the outermost Mn^III^ ions exhibit square pyramidal geometries with an axial weak bonding interaction of ~3.5 Å to a phenolato O atom. Magnetic studies reveal that the compound has an *S* = 4 ground state as a result of ferromagnetic exchange between the two antiferromagnetically coupled {${\text{Mn}}_{3}^{\text{III}}$} triangles; it is also an SMM with *U*_eff_ = ~20 cm^−1^. While the *S* and *U*_eff_ values are not spectacular, the *D*-value (−1.2 cm^−1^) certainly is.

In an elegant series of studies (Milios et al., 2007a,b,c,d, 2008), Brechin's group showed that by employing derivatised versions of the salicylaldoximate ligand (Rsao^2−^; R = Me, Et) and bulky carboxylates [R′${\text{CO}}_{2}^{-}$; R = Ph, CMe_3_, CPh_3_, Ph^2^OPh, Ph^4^OPh, PhBr, Ph(Me)_2_, 3-thiophene…], it was possible to significantly distort the core of the molecule synthesizing family members of general formula [${\text{Mn}}_{6}^{\text{III}}$O_2_(O_2_CR′)_2_(Rsao)_6_(solvents)_4−6_] in which the planarity of the {Mn^III^-N-O-Mn^III^} moieties is significantly disrupted and the carboxylate groups become monodentate. There is a dramatic effect on the magnetic properties; the ground-state spin increases from *S* = 4 to *S* = 12 and the SMM behavior improves. Studies on ~30 family members suggest that the switch from antiferromagnetic to ferromagnetic pairwise exchange occurs when the Mn^III^-N-O-Mn^III^ torsion angle becomes larger than ~31°. The more puckered the ring (-Mn-N-O-)_3_, the more ferromagnetic the exchange and the larger the barrier to magnetization reversal (*U*_eff_) becomes. These studies have allowed for a rare semi-quantitive magnetostructural correlation in SMMs which enables explanation and prediction of the magnetic properties of family members. Examples include clusters [${\text{Mn}}_{6}^{\text{III}}$O_2_(O_2_CPh)_2_(etsao)_6_(EtOH)_4_(H_2_O)_2_] (**47**; *S* = 12, *D* = −0.43 cm^−1^, *U*_eff_ = 36.9 cm^−1^), [${\text{Mn}}_{6}^{\text{III}}$O_2_{O_2_CPh(Me)_2_}_2_(etsao)_6_(EtOH)_6_] (**48**; *S* = 12, *D* = −0.43 cm^−1^, *U*_eff_ = 60.1 cm^−1^), [${\text{Mn}}_{6}^{\text{III}}$O_2_(O_2_CCMe_3_}_2_(etsao)_6_(EtOH)_5_] (**49**; *S* = 7, *D* = −0.75 cm^−1^, *U*_eff_ = 20.9 cm^−1^), [${\text{Mn}}_{6}^{\text{III}}$O_2_(O_2_CPh^4^OPh)_2_(etsao)_6_(EtOH)_4_(H_2_O)_2_] (**50**; *S* = 9, *D* = −0.37 cm^−1^, *U*_eff_ = 39.6 cm^−1^) and [${\text{Mn}}_{6}^{\text{III}}$O_2_(O_2_CPhBr)_2_(mesao)_6_(EtOH)_6_] (**51**; *S* = 11, *D* = −0.50 cm^−1^, *U*_eff_ = 34.9 cm^−1^); mesao^2−^ and etsao^2−^ are the dianions of 2-hydroxyphenylethanone oxime and 2-hydroxyphenylpropanone oxime, respectively (**L** with R = Me, Et in Figure 1). Complex **48** holds the record *U*_eff_ (60.1 cm^−1^) and *T*_B_ (~4.5 K) values for a 3d-metal cluster with SMM properties.

It should be mentioned at this point that the strategy detailed in this section is different from the approaches described in sections “Switching on” SMM Behavior through Substitution of Bridging Hydroxido Groups by End-On Azido or Isocyanato Ligands in Pre-formed Clusters, “Tweaking” the Spin, and “Switching on” SMM properties upon Conversion of Low-Spin Complexes Into High-Spin Ones Without Changing the Core. The present strategy is strictly speaking not a switching on of the SMM property (sections “Switching on” SMM Behavior Through Substitution of Bridging Hydroxido Groups by End-on Azido or Isocyanato Ligands in Pre-formed Clusters and “Switching on” SMM properties upon Conversion of Low-Spin Complexes Into High-Spin Ones Without Changing the Core), since the *S* = 4 {${\text{Mn}}_{6}^{\text{III}}$} cluster is also an SMM. Also, the increase of the total ground-state spin here is large (~200%) and not small (~20%) as in the “spin tweaking” strategy (section “Tweaking” the Spin); in both strategies the starting materials are SMMs.

### Synthesis of radical-bridged 3d-metal SMMs

As it has been clearly stated in sections Introduction and Synthesis of 3d-Metal SMMs: General Considerations, the continuing research efforts to prepare 3d-metal SMMs with higher *U*_eff_ values take into consideration the ground-state spin, *S*, and the axial zero-field splitting parameter, *D*; both these key parameters are correlated to the energy barrier height. A third important parameter, which is often overlooked, is the exchange coupling constant, *J*. The value of this physical parameter determines the separation between the spin of the ground state and the spins of the excited states. The *J*-value must be large (strong coupling) in an SMM to maintain slow magnetization relaxation at elevated temperatures and also to decrease fast magnetization relaxation through pathways involving excited spin states. An excellent synthetic strategy to achieve strong magnetic exchange is the incorporation of radical ligands into coordination complexes. The direct exchange between paramagnetic 3d-metal ions and paramagnetic radical ligands can result in very strong magnetic coupling, stronger than the more common superexchange interactions between the metal centers (Demir et al., 2015). In the coordination clusters of 3d-metal ions, the radical ligand is often bridging. Transition metal-based polynuclear SMMs possessing a radical ligand are extremely rare, most of them being dinuclear. We give an example of a dinuclear complex below.

**Figure 13:**
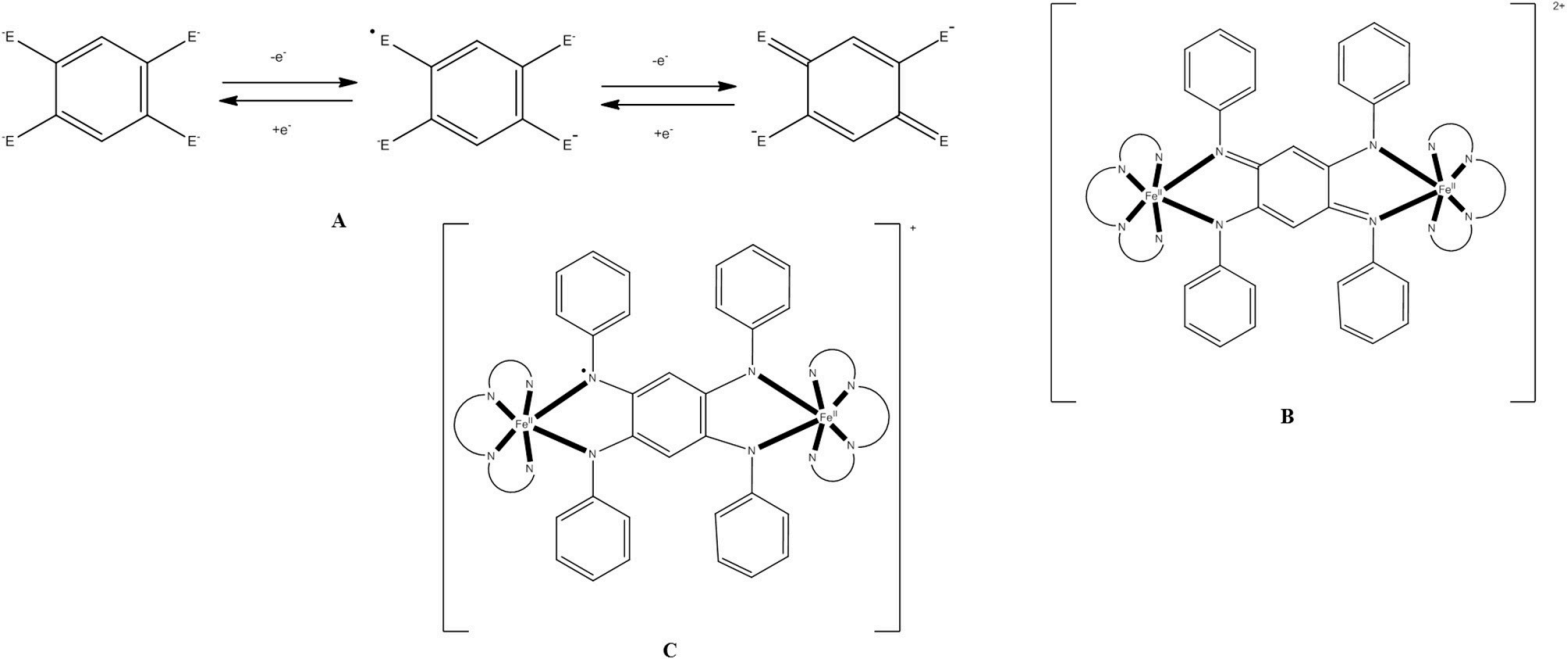
**(A)** Redox transformations of anionic benzoquinonoid ligands (E = O and NR; R is the Ph group in the present example). **(B)** Schematic representation of the molecular structure of the dication that is present in **52**. **(C)** Schematic representation of the molecular structure of the monocation that is present in **53**. N^∧^N^∧^N^∧^N^∧^ represents the tetradentate chelating ligand tpya. Coordination bonds are drawn in bold (Schematic drawing inspired by Jeon et al., 2013; Demir et al., 2015).

Benzoquinonoid groups (**A** in Figure 13) are ideal ligands for the synthesis of radical-bridged SMMs with strong magnetic coupling. They can easily undergo redox reactions providing paramagnetic and diamagnetic electron-transfer isomeric species. The reaction of Fe(CF_3_SO_3_)_2_ with tris(2-pyridylmethyl)amine (tpya; **T** in Figure 1) in anhydrous MeCN, followed by treatment with a mixture of Li{N(SiMe_3_)_2_} and ^NPh^LH_2_ (**U** in Figure 1) gives a dark brown solution, from which dark yellow crystals of the dinuclear complex [${\text{Fe}}_{2}^{\text{II}}$(^NPh^L^2−^)(tpya)_2_](CF_3_SO_3_)_2_ (**52**) are isolated, Equation (41). Subsequent reaction of this complex with a strong reducing agent, e.g., [Co^II^(C_5_Me_5_)_2_] in MeCN/MeOH at −35°C leads to the dark blue complex [${\text{Fe}}_{2}^{\text{II}}$(^NPh^L^3−•^)(tpya)_2_](CF_3_SO_3_) (**53**), Equation (42). Compound **53** is the one-electron reduced analog of **52** (Jeon et al., 2013).

(41) $$\begin{array}{l} 2\;{\text{Fe}}^{\text{II}}{\left({\text{CF}}_{3}{\text{SO}}_{3}\right)}_{2}+2\text{ tpya}+{\;}^{\text{NPh}}{\text{LH}}_{2}+2\text{ Li}\{\text{N}{\left({\text{SiMe}}_{3}\right)}_{2}\}\overset{\text{MeCN}}{\to }\;\\ \;[{\text{Fe}}_{2}^{\text{II}}{(}^{\text{NPh}}{\text{L}}^{2-}){\left(\text{tpya}\right)}_{2}]{\left({\text{CF}}_{3}{\text{SO}}_{3}\right)}_{2}+2\text{ Li}{\left({\text{CF}}_{3}{\text{SO}}_{3}\right)}_{2}\\ \;+2\text{ HN}{\left({\text{SiMe}}_{3}\right)}_{2} \end{array}$$

(42) $$\begin{array}{l} {}[{\text{Fe}}_{2}^{\text{II}}{(}^{\text{NPh}}{\text{L}}^{2-}){\left(\text{tpya}\right)}_{2}]{\left({\text{CF}}_{3}{\text{SO}}_{3}\right)}_{2}+[{\text{Co}}^{\text{II}}{\left({\text{C}}_{5}{\text{Me}}_{5}\right)}_{2}]\;\\ \;\overset{{\text{MeCN/Et}}_{\text{2}}\text{O}}{\underset{-35^{{}^{\circ}}\text{C}}{\to }}\;[{\text{Fe}}_{2}^{\text{II}}{(}^{\text{NPh}}{\text{L}}^{3-•}){\left(\text{tpya}\right)}_{2}]\left({\text{CF}}_{3}{\text{SO}}_{3}\right)\\ \;+[{\text{Co}}^{\text{III}}{\left({\text{C}}_{5}{\text{Me}}_{5}\right)}_{2}]\left({\text{CF}}_{3}{\text{SO}}_{3}\right) \end{array}$$

The molecular structures of both unreduced (**52**) and reduced (**53**) complexes show two {(tpya)Fe^II^}^2+^ subunits connected by a deprotonated azophenine bridging ligand, with two N atoms occupying *cis* sites of each octahedral Fe^II^ ion; the four N atoms of the tetradentate chelating tpya ligand complete the coordination sphere at each metal center (see **B** for **52** and **C** for **53** in Figure 13). While the gross structural features of the cations of **52** and **53** are similar, bond lengths in the backbone of the bridging ligand show key differences which permit the assignment of a trianionic bridging ligand with an unpaired electron (^NPh^L^3−•^) in the case of **53**. This assignment is supported by ^57^Fe-Mössbauer studies; the identical isomer shifts, δ, in the two complexes suggest that the reduction is ligand-centered, while the larger quadrupole splitting, Δ*E*_Q_, in **53** is due to the change of the ligand field at Fe^II^ associated with ligand reduction and the larger distortion from the ideal octahedral environment at the metal ion in **53** compared to **52**. Variable-temperature dc magnetic susceptibility studies for solid **52** show a weak antiferromagnetic exchange interaction between the high-spin Fe^II^ ions through the diamagnetic ^NPh^L^2−^ ligand leading to an *S* = 0 ground state; the *J* value is −2.90(2) cm^−1^ based on the spin Hamiltonian Ĥ = −2*J*(Ŝ_Fe1_·Ŝ_Fe2_). Data for **53** reveal an exceptionally strong antiferromagnetic exchange between Fe^II^ centers and the ^NPh^L^3−•^ radical ligand (*J* ≤ −900 cm^−1^ based on the same Hamiltonian). SMM behavior was confirmed for **53** through ac susceptometry with a *U*_eff_ value of 50 cm^−1^. The |*J*| ≥ 900 cm^−1^ value is the strongest magnetic exchange ever observed in an SMM.

### Supramolecular approaches in manganese SMM chemistry

For several technological applications of SMMs, coupling of two or more SMMs to each other or to components of a device is very important. The coupling must be very weak in order to preserve the intrinsic single-molecule properties of each SMM. This coupling can be, in principle, achieved by H bonds and many H-bonded dimers, 1D, 2D, and 3D networks have been reported in the literature. However, reliance on H bonds to obtain the desired inter-SMM associations is a synthetic weakness, because it is difficult to control the oligomerization and to achieve retention of the supramolecular structure in solution. The answer to the problem is the linking of SMMs via coordination bonds. This approach gives often coordination polymers (1D, 2D, 3D) in which the coupling between the SMMs is strong leading to loss of the SMM property and appearance of single-chain magnetism (for 1D compounds) or ordering (for the 2D and 3D compounds). A synthetic challenge is thus the connection of “good” SMMs into discrete oligomers and the achievement of very weak exchange coupling between them through the bridging ligands, so that the properties of the oligomer can be considered as a small perturbation of the properties of the constituent SMM subunits. The desired oligomers can be prepared by direct mixing simple starting materials, but such a route offers limited synthetic control and there is no guarantee that the magnetic properties will be the targeted ones. A good strategy is the use of building-block approaches to link SMMs together employing carefully chosen linker groups that will provide weak inter-SMM interactions and ensure that discrete oligomeric species (and not polymers) are obtained. The groups of Papaefstathiou, Brechin, Christou, and Escuer, among others, have successfully contributed into this general synthetic goal (Stoumpos et al., 2009; Inglis et al., 2010; Cordero et al., 2011; Nguyen et al., 2011, 2015, 2016; Mowson et al., 2013). We herein present examples of the supramolecular approaches which have been almost exclusively developed in Mn SMM chemistry.

Complexes **45a**, **45b**, and **45c** (section “Switching on” SMM properties upon Conversion of Low-Spin Complexes Into High-Spin Ones Without Changing the Core) are good SMM building blocks for the application of the supramolecular approach. They have an *S* = 6 ground state and are SMMs. Their triangular cations have C_3_ symmetry with the carboxylate and mpko^−^ ligands on opposite sites of the plane defined by the three metal ions. This tripodal arrangement of the three oximato groups suggested (Nguyen et al., 2011, 2016) that their replacement with dioximate groups to link multiple {${\text{Mn}}_{3}^{\text{III}}$} units together might give oligomeric complexes rather than polymeric species. The linker of choice has been 3-phenyl-1,5-bis(pyridin-2-yl)pentane-1,5-dione (pdpdH_2_; **V** in Figure 1). This ligand consists of two mpkoH (**H** with R = Me in Figure 1) groups that are connected by a benzyl unit. The 1:2 reaction of **44a** and pdpdH_2_ in CH_2_Cl_2_ gives complex [${\text{Mn}}_{12}^{\text{III}}$O_4_(O_2_CMe)_12_(pdpd)_6_](ClO_4_)_4_ (**54**) in 35% yield, Equation (43). The excess of pdpdH_2_ (the stoichiometric ratio is 2:3) gives the maximum possible yield.

(43) $$\begin{array}{l} 4\;[{\text{Mn}}_{3}^{\text{III}}\text{O}{\left({\text{O}}_{2}\text{CMe}\right)}_{6}{\left(\text{py}\right)}_{3}]\left({\text{ClO}}_{4}\right)+6\;{\text{pdpdH}}_{2}\;\overset{{\text{CH}}_{2}{\text{Cl}}_{2}}{\to }\\ {}[{\text{Mn}}_{12}^{\text{III}}{\text{O}}_{4}{\left({\text{O}}_{2}\text{CMe}\right)}_{12}{\left(\text{pdpd}\right)}_{6}]{\left({\text{ClO}}_{4}\right)}_{4}+12\;\left(\text{pyH}\right)\left({\text{O}}_{2}\text{CMe}\right) \end{array}$$

The cation of **54** contains 4 {${\text{Mn}}_{3}^{\text{III}}$(μ_3_-O)}^7+^ subunits connected by 6 pdpd^2−^ ligands to give a rectangular {${\text{Mn}}_{3}^{\text{III}}$}_4_ supramolecular species with each triangular unit at a corner. One of the two 2.11-${\text{MeCO}}_{2}^{-}$ groups bridging each edge of **44a** has been replaced by a bridging oximato group from a pdpd^2−^ ligand. The short and long sides of the rectangle are bridged by two and one pdpd^2−^ ligands, respectively. In addition, the 2-pyridyl N atoms of pdpd^2−^ have replaced the terminal py molecules of **44a**. The local structure of each {${\text{Mn}}_{3}^{\text{III}}$} unit of **54** is similar to that of the mpko^−^ - containing complex **45a**. As in **45a**, the 3 bridging oximato groups are on the same side of the {${\text{Mn}}_{3}^{\text{III}}$} plane, and this leads to a tetramer of triangles rather than a coordination polymer. Solid-state dc magnetic susceptibility studies show that the {${\text{Mn}}_{3}^{\text{III}}$} subunits of **54** possess an *S* = 6 ground state. Ac susceptibility studies indicate the dodecanuclear cluster to be a tetramer of {${\text{Mn}}_{3}^{\text{III}}$} SMMs. Magnetization vs. dc field studies on a single crystal of **54**·xCH_2_Cl_2_ give hysteresis loops below 1 K that exhibit exchange-biased QTM with a bias field of 0.19 T. Simulation of the loops reveals that each {${\text{Mn}}_{3}^{\text{III}}$} subunit is exchange-coupled to the two neighboring subunits connected to it by the pdpd^2−^ bridging ligands, with an antiferromagnetic inter-{${\text{Mn}}_{3}^{\text{III}}$} exchange interaction of *J*/*K*_B_ = −0.008 cm^−1^. Thus, the synthesis of **54** confirms the feasibility of connecting multiple Mn_x_ SMMs to isolate a discrete supramolecular “cluster of SMMs” with only weak coupling between them.

Supramolecular aggregates of the {${\text{Mn}}_{3}^{\text{III}}$} SMMs **45** can also be prepared through the carboxylato sites of the staring materials, thus complementing the reports of aggregation at the oximato positions using dioximate linkers (Mowson et al., 2013). The latter strategy is not by substitution on pre-formed **45**, but the former is. The 2:3 reaction of **45a** and fumaric acid (fumH_2_; **W** in Figure 1) in MeCN gives complex [${\text{Mn}}_{12}^{\text{III}}$O_4_(fum)_6_(mpko)_12_](ClO_4_)_4_ (**55**) in ~35% yield, Equation (44). The reaction of **45a** and bulky α-truxillic acid (atxH_2_; **X** in Figure 1) in a 2:3 ratio in MeCN/EtOH leads to cluster [${\text{Mn}}_{6}^{\text{III}}$O_2_(atx)_4_(mpko)_6_] (**56**) in 30% yield, Equation (45).

(44) $$\begin{array}{l} 4\;[{\text{Mn}}_{3}^{\text{III}}\text{O}{\left({\text{O}}_{2}\text{CMe}\right)}_{3}{\left(\text{mpko}\right)}_{3}]\left({\text{ClO}}_{4}\right)+6\;{\text{fumH}}_{2}\overset{\text{MeCN}}{\to }\\ {}[{\text{Mn}}_{12}^{\text{III}}{\text{O}}_{4}{\left(\text{fum}\right)}_{6}{\left(\text{mpko}\right)}_{12}]{\left({\text{ClO}}_{4}\right)}_{4}+6\;{\text{MeCO}}_{2}\text{H} \end{array}$$

(45) $$\begin{array}{l} 2\;[{\text{Mn}}_{3}^{\text{III}}\text{O}{\left({\text{O}}_{2}\text{CMe}\right)}_{3}{\left(\text{mpko}\right)}_{3}]\left({\text{ClO}}_{4}\right)+4\;{\text{atxH}}_{2}\overset{\text{MeCN}/\text{EtOH}}{\to }\\ {}[{\text{Mn}}_{6}^{\text{III}}{\text{O}}_{2}{\left(\text{atx}\right)}_{4}{\left(\text{mpko}\right)}_{6}]+6\;{\text{MeCO}}_{2}\text{H}+2\;{\text{HClO}}_{4} \end{array}$$

**Figure 14:**
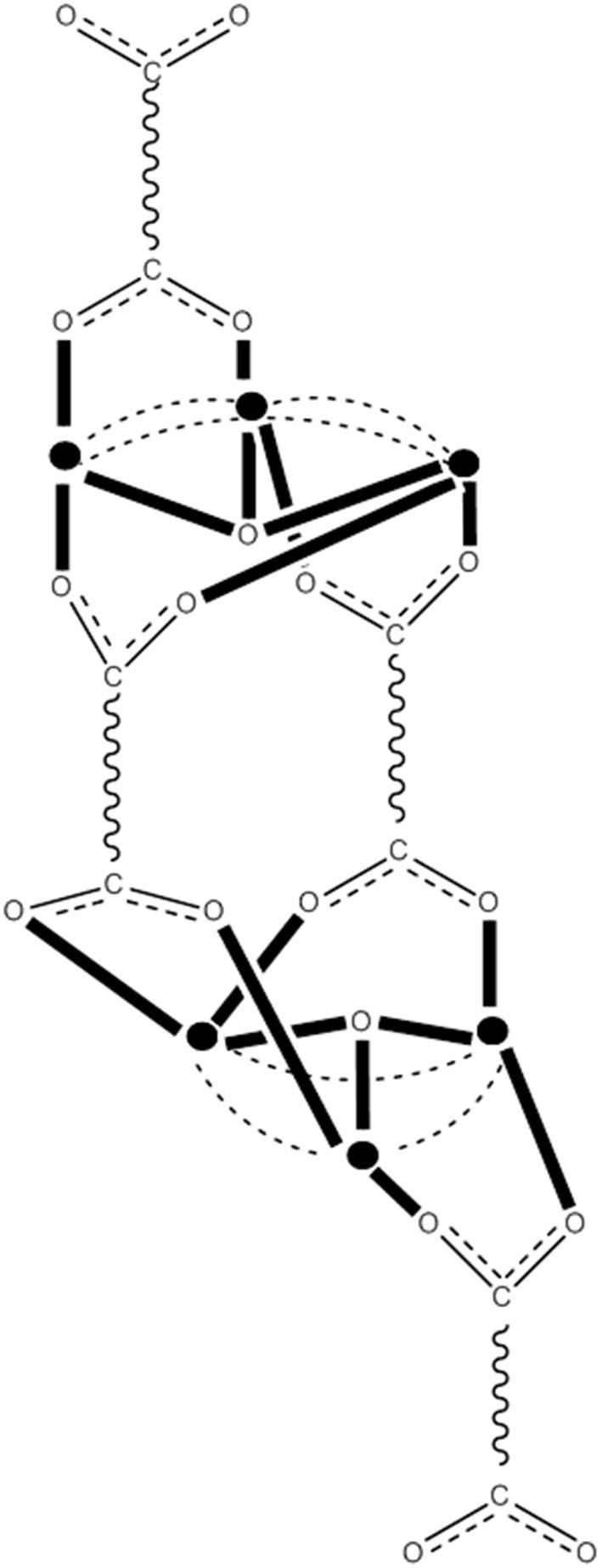
An extended view of the core of cluster **56**. The symbol 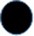 represents Mn^III^. The dashed curve represents the diatomic oximato groups of mpko^−^. The line 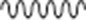 represents the aromatic and aliphatic backbone of the atx^2−^ ligands. Coordination bonds are drawn in bold (Schematic drawing inspired by Mowson et al., 2013).

The cation of **55** consists of a {${\text{Mn}}_{3}^{\text{III}}$}_4_ tetrahedron with a 4.1111 fum^2−^ (**TT** in Figure 2) bridging each edge to give an adamantane-like motif, which is a common unit in many supramolecular systems. Each carboxylate group of fum^2−^ ligand is 2.11 and thus bridges within a {${\text{Mn}}_{3}^{\text{III}}$} subunit like the ${\text{MeCO}}_{2}^{-}$ group that it replaces. Cluster **55** can be described as a tetramer of **45a** held together by fum^2−^ linkers. Complex **56** is a {${\text{Mn}}_{3}^{\text{III}}$}_2_ dimer (see Figure 14 for its core); for steric reasons (two bulky Ph substituents per ligand) it is bridged by only two 4.1111 atx^2−^ groups (**UU** in Figure 2), with two additional atx^2−^ groups coordinated only through one carboxylate at each {${\text{Mn}}_{3}^{\text{III}}$} subunit (**VV** in Figure 2). The mpko^−^ ligands in **55** and **56** bind in the familiar 2.111 mode (**MM** with R = Me and M = Mn^III^ in Figure 2). Detailed magnetic studies indicate that the *S* = 6 {${\text{Mn}}_{3}^{\text{III}}$} subunits within the “tetramer” (**55**) and “dimer” (**56**) aggregates are only weakly interacting and retain the intrinsic SMM properties of the “monomeric” {${\text{Mn}}_{3}^{\text{III}}$} complex (**45a**). Ac magnetic susceptibility studies of **55** and **56** show the appearance of out-of-phase signals below 3.0 K, indicating the slow relaxation of SMMs.

Another attractive ligand for the supramolecular synthesis of Mn SMM is 1,3-di(pyridin-2-yl)propane-1,3-dione dioxime (dpdH_2_; **Y** in Figure 1). This ligand represents a fusion of two mpkoH (**H** with R = Me in Figure 1) groups at the methyl group. The single sp^3^ C atom at the center of the molecule reduces the conformational flexibility with respect to pdpdH_2_ (**V** in Figure 1), and the combination of a ~109° ditopic dioximate with a tritopic {${\text{Mn}}_{3}^{\text{III}}$} unit should yield a {${\text{Mn}}_{3}^{\text{III}}$}_2_ “dimer” with three linker groups and parallel {${\text{Mn}}_{3}^{\text{III}}$} planes (to facilitate study of quantum properties). Another parameter for the selection of dpdH_2_ is the fact that binding by tridentate 2-pyridyloximate groups of three doubly deprotonated linkers would provide robustness and rigidity in the “dimer,” resulting in retention of the structure in solution. Moreover, the single sp^3^ carbon atom was expected to propagate a very weak (but non-zero) superexchange interaction between the two {${\text{Mn}}_{3}^{\text{III}}$} SMMs and therefore a small perturbation on a two-SMM system (Nguyen et al., 2015). The reaction of **44a** with dpdH_2_ and I_2_ in a 2:3:6 molar ratio in EtOH/CH_2_Cl_2_ gives [${\text{Mn}}_{6}^{\text{III}}$O_2_(O_2_CMe)_6_(dpd)_3_](I_3_)_2_ (**57**) in 22% yield, Equation (46). I_2_ is added to stop reduction of some of the Mn^III^ to Mn^II^, as sometimes observed in such reactions.

(46) $$\begin{array}{l} 2\;[{\text{Mn}}_{3}^{\text{III}}\text{O}{\left({\text{O}}_{2}\text{CMe}\right)}_{6}{\left(\text{py}\right)}_{3}{]}^{+}+3\;{\text{dpdH}}_{2}\overset{\text{EtOH}/{\text{CH}}_{2}{\text{Cl}}_{2}}{\to }\\ {\left[{\text{Mn}}_{6}^{\text{III}}{\text{O}}_{2}{\left({\text{O}}_{2}\text{CMe}\right)}_{6}{\left(\text{dpd}\right)}_{3}\right]}^{2+}+6\;{\text{pyH}}^{+}+6\;{\text{MeCO}}_{2}^{-} \end{array}$$

The structure of the hexanuclear dication consists of two {${\text{Mn}}_{3}^{\text{III}}$(μ_3_-O)}^7+^ subunits linked by three dpd^2−^ ligands to give a {${\text{Mn}}_{3}^{\text{III}}$} “dimer,” with the two {${\text{Mn}}_{3}^{\text{III}}$} planes being parallel. Each trinuclear subunit is structurally similar to that of “monomer” **45a**. The dianionic ligand adopts the 4.111111 coordination mode (**WW** in Figure 2), each 2-pyridyloximate subgroup bridging two Mn^III^ ions belong to a {${\text{Mn}}_{3}^{\text{III}}$} subunit. Solid-state dc and ac magnetic susceptibility studies indicate that each {${\text{Mn}}_{3}^{\text{III}}$} subunit of the “dimer” is a separate SMM with an *S* = 6 ground state and that the two SMM subunits interact very weakly in a ferromagnetic fashion. The ferromagnetic nature of the coupling arises from a spin polarization mechanism through the central sp^3^ carbon atom, caused by spin density delocalized into the dpd^2−^ ligand π systems from the Mn^III^ d_π_ magnetic orbitals. High-frequency EPR spectra on a single crystal of **57** exhibit signal splittings that indicate quantum superposition of two SMMs. Studies on toluene/MeCN (1:1 v/v) solutions display the same spectra, indicating that the “dimer” retains its structure and the weak inter-{${\text{Mn}}_{3}^{\text{III}}$} coupling in solution. This is of particular importance in the currently intense efforts for the deposition of SMMs on surfaces and other substrates.

## Brief summary and outlook

We hope that this review has provided the readers with a “flavor” of the synthetic chemistry and reactivity studies of 3d-metal coordination clusters with SMM properties. Empirical routes (section The Years of Innocence: “Try and See” Exercises) and strategies (section The Years of Design: Strict and Less Strict Synthetic Strategies) have been discussed and critically analyzed through selected examples; often the distinction between an empirical route and a strict strategy is blurred. Particular emphasis has been given in the synthetic rationale behind the reactions and in the criteria for the selection of the metal ions and the ligands. Most strategies refer to Mn chemistry, because the relatively large number of unpaired electrons (four) and the large easy-axis anisotropy (negative *D*) for the Jahn-Teller elongated high-spin Mn^III^ make this ion an ideal candidate for the construction of SMMs.

The extremely low temperatures at which the polynuclear 3d-metal complexes operate as SMMs (<4.5 K) make these molecules not suitable for technological applications. Their future utility is in low-temperature and specialized applications that take full advantage of their unique molecular characteristics (size, monodispersity, crystallinity, good solubility, feasibility of altering their peripheral ligation, and well-defined quantum properties). However, the inability of polynuclear 3d-metal SMMs for every day applications does not underestimate their importance. These compounds are responsible for great revolutions in synthetic (for their preparation and reactivity) and physical (for their characterization and study) Inorganic Chemistry, a field that has undergone a second renaissance (the first was in the 50's and early 60's) after the second world war.

Most research efforts in the chemistry of molecular compounds exhibiting slow magnetization relaxation (at zero field) are now focused on 4f- and 5f-metal SIMs for reasons outlined in section Introduction. However, we do believe that there is still enough room for the improvement of the existing strategies and the development of new ones in the chemistry of 3d-metal clusters with SMM properties. We expect that the synthesis and the study of radical-bridged 3d-metal SMMs (section Synthesis of Radical-Bridged 3d-Metal SMMs) will be intensified in the future because of the potential of huge *J*-values. We also predict new developments in the supramolecular chemistry of 3d-metal SMMs (section Supramolecular Approaches in Manganese SMM Chemistry), since this is related to the linking of SMMs on surface and other substrates. A final point of synthetic interest is that the larger radial extension of the 4d and 5d orbitals (compared to the 3d ones) allows for stronger exchange coupling through diamagnetic bridging ligands. Since the strong exchange is a key factor in SMM research, we anticipate an increased synthetic activity in the area of 4d- and 5d-metal cluster SMMs (not covered in this review).

We conclude by pointing out that it is hoped that this review will be proven useful for inorganic chemists who are already active in SMM research or just enter into this area, and we shall be happy if the readers enjoy the review as we enjoyed writing it.

## Author contributions

DM and EP jointly studied the literature, proposed some of the cited examples, prepared the figures, and typed the manuscript. SP developed the concept of the review and wrote the manuscript.

### Conflict of interest statement

The authors declare that the research was conducted in the absence of any commercial or financial relationships that could be construed as a potential conflict of interest.
